# COVID-19 Vaccination and Mental Stress within Diverse Sociodemographic Groups

**DOI:** 10.3390/ijerph191912932

**Published:** 2022-10-09

**Authors:** Wasiq Khan, Bilal M. Khan, Salwa Yasen, Ahmed Al-Dahiri, Dhiya Al-Jumeily, Khalil Dajani, Abir Hussain

**Affiliations:** 1 School of Computer Science and Mathematics, Liverpool John Moores University, Liverpool L3 3AF, UK; 2 Department of Computer Science and Engineering, California State University, San Bernardino, CA 92407, USA; 3 The Hollies Family Surgery, 10 Elbow Lane, Formby, Liverpool L37 4AF, UK; 4 Norwood Surgery, Norwood Ave., Southport PR9 7EG, UK; 5 Department of Electrical Engineering, University of Sharjah, Sharjah P.O. Box 27272, United Arab Emirates

**Keywords:** mental stress dataset, COVID-19 mental health issues, vaccine dataset, vaccine sociodemographic, vaccine acceptance rate, future epidemics

## Abstract

In this study, we surveyed 635 participants to determine: (a) major causes of mental stress during the pandemic and its future impacts, and (b) diversity in public perception of the COVID-19 vaccination and its acceptance (specifically for children). Statistical results and intelligent clustering outcomes indicate significant associations between sociodemographic diversity, mental stress causes, and vaccination perception. For instance, statistical results indicate significant dependence between gender (we will use term ‘sex’ in the rest of the manuscript) and mental stress due to COVID-19 infection (*p* = 1.7 × 10^−5^). Over 25% of males indicated work-related stress compared to 35% in females, however, females indicated that they were more stressed (17%) due to relationships compared to males (12%). Around 30% of Asian/Arabic participants do not feel that the vaccination is safe as compared to 8% of white British and 22% of white Europeans, indicating significant dependence (*p* = 1.8 × 10^−8^) with ethnicity. More specifically, vaccination acceptance for children is significantly dependent with ethnicity (*p* = 3.7 × 10^−5^) where only 47% participants show willingness towards children’s vaccination. The primary dataset in this study along with experimental outcomes identifying sociodemographic information diversity with respect to public perception and acceptance of vaccination in children and potential stress factors might be useful for the public and policymakers to help them be better prepared for future epidemics, as well as working globally to combat mental health issues.

## 1. Introduction

Coronavirus disease which resulted in the 2019 pandemic, hence the name COVID-19, has infected more than 19 million people in the UK and more than 600 million worldwide as of September 2022 causing around 6,510,139 deaths [1]. The majority of the people experience mild symptoms, however there is a large number of patients who develop severe symptoms related to respiratory issues and required hospitalisation [2].

COVID-19 also known as severe acute respiratory syndrome coronavirus 2 (SARS-CoV-2) and is a new strain of coronavirus that has not been known to humans previously [3]. During 2020, COVID-19 caused significant interference to daily life because of the unavailability of the required vaccine or known treatment strategies. Governments around the world have been introducing and depending upon various policies such as quarantine measures and social distancing to control the infection and hence support healthcare systems [4].

The deterioration of economies as well as the isolation that resulted from the pandemic have inevitably increased stress among populations [5]. Due to living with the fear of being infected and death because of SARS-CoV-2 among people, researchers have indicated that many psychological problems are likely to be increased, including depression, panic, and anxiety [6,7,8]. In addition, changes in people’s work pattern, environments, and lifestyle have significant effects on mental health [9] and could have negative effects such as reduced work abilities [10]. As such, COVID-19 could be linked with various stress responses known as COVID-19 stress syndrome in which the COVID-19 stress scale (CSS) has been utilised to measure its severity and was introduced by Taylor et al. [11]. The authors assured its validity and reliability using USA and Canadian data samples. This measure is defined in five factors, including fears of socioeconomic consequences, danger and contamination fears, compulsive checking and reassurance seeking, traumatic stress symptoms, and xenophobia. The authors concluded that with the end of this pandemic, various mental health requirements will have emerged within the society.

Recently, [12,13,14] highlighted the mental stress due to COVID-19 and pandemic challenges. Likewise, [15] addressed the mental stress issues mainly due to vaccination. These works have recommended various treatments for the mental health issues mainly occurring due to the pandemic and other related factors. However, these studies focus on the vaccination- and pandemic-related mental health problems while are lacking in-depth analysis of sociodemographic diversity aspects that might be interesting to be analysed for the understanding of the variations in different sub-groups.

While the long-term solution for an effective reduction in the number of cases and deaths due to SARS-CoV-2 is to provide a globally accessible COVID-19 vaccination programme [16], it should be noted that such solution can only be effective when it is widely used and accepted, allowing global immune protection. Studies have been reporting the vaccination hesitancy which causes major issue to the successfulness of the governments’ vaccination programs. Hesitancy to be vaccinated and its effects on people’s acceptance have been attributed to various factors, including demographic, socioeconomic, and religious beliefs [17]. Reiter et al. [18,19] indicated that most of the participants are willing to get the vaccination, while Malik et al. [20] indicated differences in the acceptance of the vaccination as a result of the demographics and geographic diversity. Similar outcomes are also reported by Antoinette et al. [17] from the US as well as Kumari et al. [21] from India, while Maraqa et al.’s [22] survey on healthcare workers in Palestine indicated that only 37% of the participants are intending to get the vaccination.

Another important aspect related to vaccination is its perception within the public. Studies have found that those who consider COVID-19 as a threat engaged more with the efforts to prevent being affected by the disease, including social distancing, handwashing and wearing masks [23]. Malik et al. [20] investigated the relationship between the risks of disease perceived by the individual and their willingness to get the vaccination. Their analysis indicated that those who rate the disease as having high risks are most likely to get the vaccination. Likewise, Glöckner et al. [24] surveyed a German population and reported that those who consider there to be a high likeliness of being infected by COVID-19 are most likely to be vaccinated. Similar work is carried out by Adams et al. [25] who surveyed 5082 young adults in the age range of 18 to 25 with the intention of receiving the COVID-19 vaccination. Their results indicated that the majority of unvaccinated participants indicated willingness to be vaccinated, while those who declined to be vaccinated reported various causes for rejection, such as the desire to wait and monitor vaccination safety, concern due to side effects, and the thoughts that others could need the vaccination more. However, it would be useful to further investigate the demographical distributions of vaccination acceptance and perception, specifically its acceptance for children, which is lacking in the existing works. While these studies highlight the vaccination acceptability and perception as well as mental health issues, these works do not address the causes of contradicting conclusions with limited focus on sociodemographic diversity that might be one of the major factors to varying outcomes, as explored in the proposed study.

Research studies have also been reporting a variety of side effects due to COVID-19 vaccination, mainly including tiredness, pain in muscles, fever, fatigue, headache, tenderness, and chills [26]. Quiroga et al. [27] investigated the side effects of COVID-19 vaccination with 708 nephrologist participants. The major side effects reported included local reaction, myalgia, tiredness, and headache (representing 34%). Likewise, several works have also been addressing the mental stress and mental health issues due to the pandemic on children. For instance, a report published by UNICEF in 2020 indicated that there are possible implications of indirect and mental health issues as a result of closing the schools during the pandemic [28]. Similarly, several studies indicated that young children revert to the extensive use of electronic gadgets, sleep pattern disorder, and unhealthy and poor diets, which could lead to post-traumatic stress disorder [29,30,31]. A recent study by Agarwal [32] reported the impact and stress of COVID-19 in Indian society. The author indicated that the healthcare workers, including nurses and doctors, are on a continues frontline fight due to mass testing, caring for patients admitted to hospitals and the extra unpaid working hours. While students face stress and anxiety measures due to the worries about completing their degrees and online examinations, the authors in [32] also reported that farmers are the most hit by the pandemic since the ongoing lockdown measures made it difficult to transport their harvest crops to the local markets and hence their savings were constantly shrinking.

Table 1 summarises recent works addressing the mental health issues in relation to the COVID-19 pandemic [12,13,14,33,34], and vaccination [15,35,36]. It can be noticed that despite the fact that existing studies have been investigating various aspects related to mental stress during and after the COVID-19 pandemic and its vaccination, several limitations exist within these works, particularly publicly available datasets and survey responses (i.e., most of the datasets are not publicly available), limited aspects of sociodemographic diversity (e.g., ethnic backgrounds, sex, profession, age group, etc.), mental stress causes within subgroups, and most importantly, the coping strategies used by the public to reduce the mental health problems. Likewise, vaccination perception and acceptance (mainly for the children) with respect to varying sociodemographic factors and other social and psychological aspects (e.g., use of social media, online shopping, etc.,) are missing in most of the existing works which are addressed in the proposed study. Furthermore, it is important to note that almost all of the aforementioned literature and recent works summarised in Table 1 utilise conventional statistics and descriptive analyses that have several limitations when analysing the multidimensional datasets (e.g., the dataset in this study). The traditional statistical approaches, for instance, may provide one-to-one associations between two factors (e.g., mental stress in this case), however, they lack the analysis of simultaneous (or combined) interaction of multiple attributes. Likewise, the visual representation of complex patterns is impractical using conventional approaches (e.g., correlation, MLR, etc.,). In contrast, the proposed work overcomes these limitations by utilising intelligent clustering and visualisation techniques for a better understanding of the complex patterns and associations within the high-dimensional data. Furthermore, the availability of a public dataset in our work might be very useful for the stakeholders, policymakers, academics (e.g., students learning and practicing pattern analysis within multi-type attributes and clustering tasks), researchers, and the public, for the in-depth understanding of the diversity in public responses as well as effective future plans to combat such challenges effectively.

Considering the aforementioned limitations of the existing literature, the proposed study is first of its kind in the UK (to the best of authors’ knowledge) to conduct experiments using intelligent clustering algorithms and statistical tools to investigate the following research questions (RQ):

**RQ_1_:**  *What are the major causes of mental stress during the COVID-19 period and how do they affect people within diverse sociodemographic backgrounds?*

**RQ_2_:**  *How is vaccination perceived and accepted for families and children in the public with diverse sociodemographic attributes?*

The major contributions of the proposed work includes: (a) a primary dataset comprising a variety of aspects mainly related to the major causes of mental stress due to the COVID-19, pandemic, vaccination, and its acceptability and perception within diverse sociodemographic groups; (b) multi-attribute analysis to investigate the dependence between sociodemographic factors, vaccination types, acceptability for children, and mental stress causes; (c) utilisation of intelligent visualisations beyond the conventional statistical tools to investigate the complex relationships within the multi-attribute dataset; and (d) reporting the effective strategies used by the participants in relation to coping with the mental health issues during and after the COVID-19 period.

## 2. Materials and Methods

**Figure 1:**
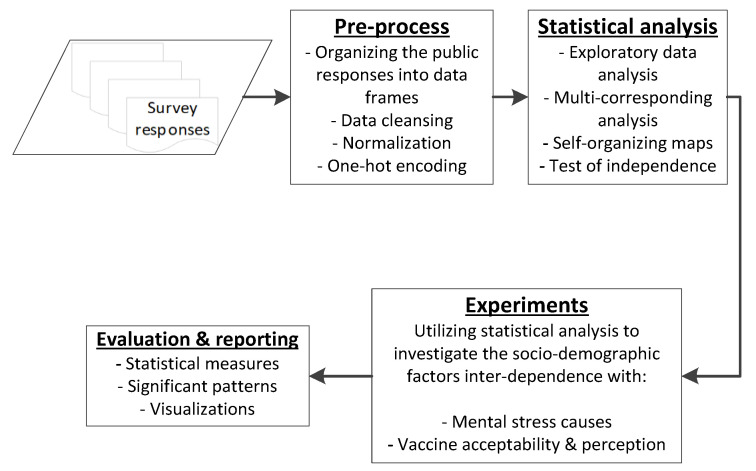
Sequential procedures used in the proposed study to analyse the sociodemographic diversity inter-dependence with mental stress causes and vaccine perception while utilising multiple statistical analysis and pattern-identification tools.

The proposed research mainly focuses the mental stress analysis, perceptual and social aspects (along with the vaccination acceptance for children) with respect to the COVID-19 pandemic and vaccination while utilising a primary dataset comprising sociodemographic diversity within the UK. Figure 1 demonstrates the sequential processing of the survey responses used in this study to investigate the proposed RQs. In the first step, the primary data are acquired through online public surveys which are then organised into the required form and forwarded to multiple pattern-matching and association identification algorithms to investigate the RQs set in this study. In the next step, comprehensive multi-attribute analysis and visualisations are performed using statistical tools, for discrete-level investigation of complex patterns within the data, inter-relationships between multiple attributes, and lower-dimensional visualisations of relationships that are easily understandable by humans. Detailed description of procedures used in each component are described in the following sections.

### 2.1. Primary Dataset Acquisition

**Figure 2:**
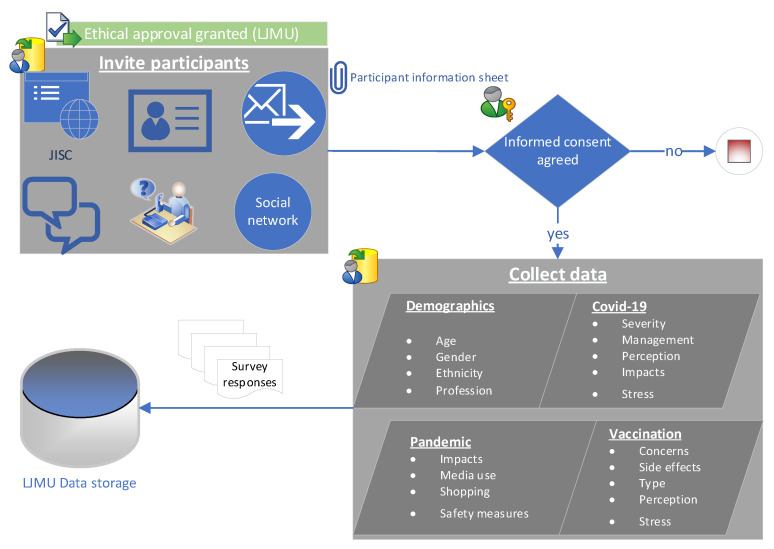
Sequential procedure for primary data collection, ethical process, survey contents, and information storage.

We present a primary dataset collected from 635 participants using online surveys. The information is collected following the ethical approval from Liverpool John Moore University (*Ethical approval reference: 21/CMP/002*). Participants are approached using random sampling via online platforms and social networks. Figure 2 demonstrates the sequential workflow of data collection in the proposed study in which, following the ethical approval, participants were invited via social and academic networks. All participants agreed to informed consent before proceeding to survey questions. The survey questions are structured following the recommendations of clinical experts and academic researchers. The questionnaire comprises four major aspects that include information about sociodemographic aspects, the COVID-19 pandemic and vaccination. The aim is to collect information from randomly selected adult participants in relation to the major causes of mental stress and social challenges due to the COVID-19 pandemic and vaccination while considering sociodemographic diversity. Furthermore, we gathered information about vaccination perception and acceptability relating more specifically to children, side effects with respect to various factors such as type of vaccine, prior COVID-19 infections and other illnesses, etc. In addition, several other social aspects are collected, including mental stress recovery, social activities, social media interaction, online shopping, and safety measures, etc., which might be useful for the related community and global policymakers. The survey data is stored on LJMU secure data repository.

Table 2 summarises the distributions of responses for the major survey questions in relation to sociodemographic factors, vaccination, the pandemic, and COVID-19. Complete set of questions and histograms of corresponding responses are presented in Supplementary S2 while the dataset (in CSV format) is presented in Supplementary S3. The collected dataset contains 635 unique responses comprising sufficient representations for sex, profession, ethnicity, and normally distributed age groups. Over 67% of participants feel they are stressed due to the impacts of COVID-19 on various aspects of their routine life. The major causes of mental stress reported include work, the pandemic, COVID-19 infection, childcare and school closures, and the negative impact on relationships. On the other hand, common activities reported to cope with mental stress include speaking to family and friends, watching TV and movies, engagement in hobbies, sports activities and use of social media. While 47% of participants think that vaccination reduced their mental stress, 33% reported no reduction, while 20% did not know about this. It is also important to note that most of the participants responded positively to vaccine acceptance for themselves and their family (75% and 80%, respectively), however, the acceptance rate reduced substantially to 47% for children while 5% indicated no acceptance at all. The common vaccination side effects include tiredness, muscle pain or swelling, headache, aches or chills, fever, and nausea. Furthermore, we asked for additional information (i.e., survey questions) that include social interactions, future safety measures, previous illnesses, online shopping, COVID-19 severity, and management. The variations in responses presented in Table 2 and the complex relationships between different aspects including sociodemographic diversity, vaccination and COVID-19 attributes are further investigated in the following sections.

### 2.2. Data Preparation

Public responses per question are transformed into data frames comprising questions as attributes (i.e., columns in Table 2) and user responses as data samples (see the dataset file in Supplementary S3). The dataset contains binary (yes/no), ordinal (e.g., age group), and multi-nominal (e.g., profession) attributes. To employ the statistical and visualisation tools effectively, we transformed the dataset into the specifically required form, the multi-choice questions such as side effects where the user can select more than one option. For this purpose, we employed one-hot encoding (i.e., dummy coding) to transform the multi-choice and multi-nominal attributes into binary categories where appropriate. For example, stress-Cause in Table 2 comprising 9 categories can be transformed into 9 binary attributes with 1 and 0 representing presence (yes) and absence (no), respectively. The processed dataset is free of errors, missing values and is in the required form which is then forwarded to multiple pattern-matching and association identification algorithms to investigate the RQs set in this study.

### 2.3. Statistical Analysis and Visualisations

Based on the proposed RQs and type of dataset, we employ multi-correspondence analysis (MCA), test of independence (i.e., Chi-square test), and self-organising maps (SOM), to analyse the complex relationships between multiple attributes and visualise the patterns in lower-dimensional space. MCA is one of the most powerful exploratory multivariate tools to visualise the inter-relationships between multiple categorical attributes. Technically, MCA uses the standard correspondence analysis to produce proximities in a low-dimensional map. The eigenvalues generated through single-value decomposition can be used to identify the principal components that are used to visualise the attributes’ correspondence in a lower-dimensional map. We employed the ‘contrib measure’ representing the variable categories contributing to each dimension of MCA. A larger ‘contrib’ value indicates better explaining (or defining) the variability in the dataset for that dimension and vice versa. Further information about mathematical formulation and applications of MCA can be found elsewhere [38].

While the MCA are useful to show the correspondence between multiple attributes, the simultaneous visualisation of all principal components is impractical for humans’ visual interpretations. For this purpose, we employ the supervised self-organising maps (SOM) which are established for their powerful visualisations of multidimensional data and complex patterns in lower dimensions (usually, two-dimensional) maps. The topological properties of the input data are preserved within the competitive learning used by SOM in contrast to error minimisation approaches in other types of neural networks. Data samples (i.e., participants’ responses in our study) are recursively projected to SOM to determine the best matching unit (i.e., winning neuron) based on the distance from its weights and the input sample (i.e., one row from the dataset). The weight update is performed for a predefined neighbourhood radius which results in grouping of similar samples within the winning neuron and its neighbourhood neurons using:

w_j (n + 1) = w_j (n) + η(n) h_ji(x) (n)〖(x(n) − w〗_j (n))(1)

where, η(n) is the learning rate and h_ji(X) (n) is the neighbourhood function around the winner neuron i(x) in equation (1). Both, η(n) and h_ji(X) (n) vary dynamically to achieve optimal results. While the dataset is in categorical form, the one-hot encoding of attributes preserves the numerical property of data as required for the competitive learning-based clustering algorithms. Further explanation and mathematical formulation of SOM can be found elsewhere [38].

## 3. Results and Discussions

Experiments are conducted using the processed dataset, statistical tools, and visualisation algorithms to investigate the outlined RQs and corresponding hypothesis. It can be noticed that the dataset covers a variety of other aspects (see Supplementary S2) in addition to the attributes presented in Table 2 which are beyond the scope of the analysis presented in this study. We mainly focus on two major research aspects in this work (see RQs) to investigate the dependence between sociodemographic diversity, major causes of mental stress, vaccination perception, and its acceptability. Detailed statistical outcomes and visualisations for each RQs are presented as follows.

### 3.1. Mental Stress Causes

Various causes of mental stress are reported by the participants (see Table 2) mainly including work 48 (%), the pandemic (43%), COVID-19 infection (36%), childcare and school closure (25%), and relationships (22%). However, it would be useful to investigate the variations within the public responses with respect to sociodemographic diversity (such as sex, ethnic background, etc.). To analyse the variation in mental stress causes with respect to sociodemographic diversity, we set up following hypotheses:

**H_0__1.**  *Sociodemographic diversity is independent of mental stress causes due to COVID-19 infection and its effects.*

**H_1__1.**  *Sociodemographic diversity is dependent of stress causes due to COVID-19 infection and its effects.*

#### 3.1.1. Sex (Male, Female)

Existing studies, such as [39,40], reported the significant differences in COVID-19 infection and mortality rate across genders. Males are more affected as compared to females [38,40]. However, these works lack the investigation of differences between males and females with respect to major stress causes, such as the pandemic, COVID-19, and vaccinations effects, etc.

**Figure 3:**
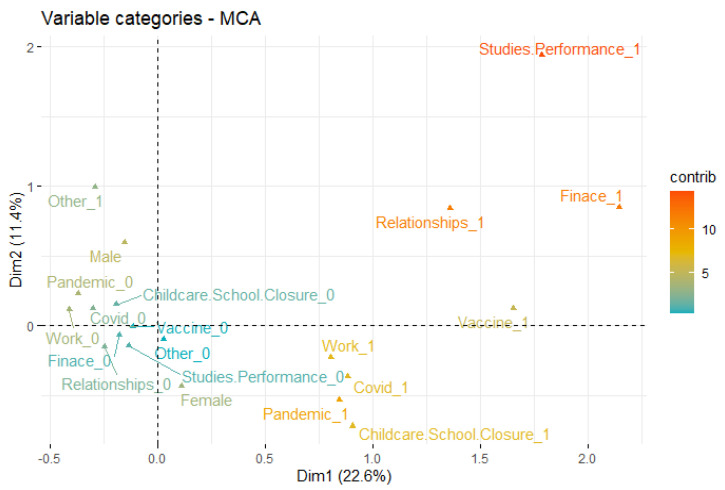
MCA outcomes (first two dimensions) indicating sex correspondence to major stress causes reported in public responses. The closer the attributes, the higher the correspondence and vice versa. Green to red ‘contrib’ colour scale indicates the contribution level being low to high, respectively, for corresponding dimension of MCA.

Figure 3 shows the first two dimensions of MCA retaining 34% of the total inertia (variation) contained in the data. The variations between the male (top left quadrant) and female (bottom right) groups indicate the difference in responses as well as relationships to mental stress causes. For instance, working females indicated more stress due to several factors such as school closures and the pandemic, as compared to male participants who correspond to ‘other’ factors. It can also be observed that work, COVID-19 (infection), the pandemic, and child school closure are very close to each other within the plot (Figure 3) which shows their inter-relationships as well as the correspondence to sex (and mainly females). This aligns with the recent findings from [13,33,34] reporting females to be more psychologically distressed during the pandemic. Likewise, childcare and school closure outcomes in Figure 3 also endorse findings from another recent work [12] which indicated that the majority of the parents are ‘being stressed’ by the closure of schools and childcare facilities.

**Figure 4:**
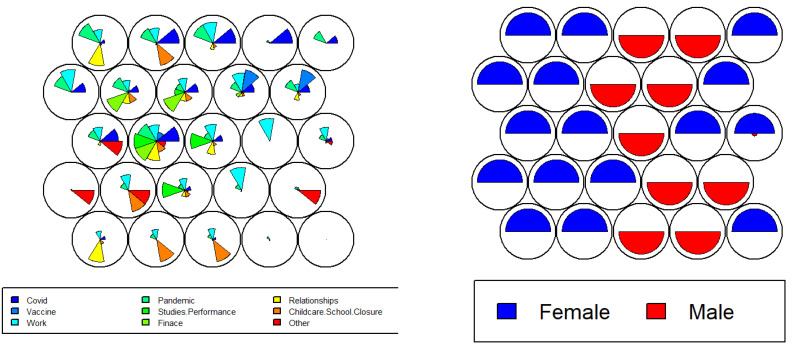
SOM code plot for two-dimensional visualisation of inter-relationships between multiple causes of mental stress (**left** side plot) within the dataset and participants’ sex (**right** side plot).

Despite MCA outcomes in Figure 3 providing better visualisations of the variables’ distributions and correspondence, we require additional dimensions to perfectly represent that data which is impractical with visualisation tools used in most of the literature and MCA. Alternatively, SOM provides an efficient way to represent multiple factors as well as corresponding distributions useful to analyse the inter-relationships and complex patterns as shown in Figure 4. It can be observed that the ‘relationships’ attribute indicates high occurrences in the female group as compared to males, while COVID-19, the pandemic and work appeared regardless of sex. The childcare/school closure indicated high correlation with females compared to males.

Furthermore, the SOM heatmap produces individual relationships between these attributes as shown in Supplementary S1 (see Figure S1). It clearly indicates the high appearances of child school closure (left side of the heat map) and relationships within females as compared to male groups. On the other hand, both groups indicated similar behaviour for being mentally stressed by COVID-19, the pandemic and work factors. These findings are also partially endorsed by the existing works [12,13], however, the proposed outcomes provide discrete-level information within the subgroups (e.g., males vs. females) along with the lower-dimensional representation of high-dimensional data (e.g., Figure 3 and Figure 4). Overall, the above findings clearly indicate the significant associations between sex (i.e., male/female) and factors causing the mental stress.

#### 3.1.2. Age Groups

**Figure 5:**
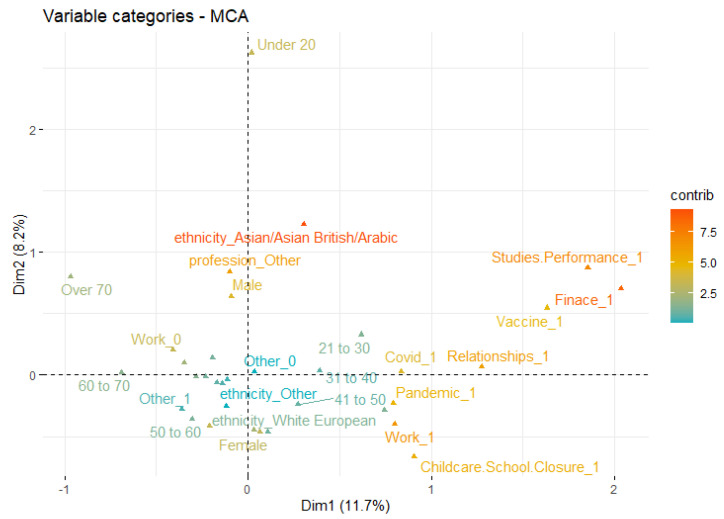
MCA outcomes (first two dimensions) for the combined visualisation of major stress causes and sociodemographic attributes (ethnicity, profession, sex, and age groups).

Similar to the different COVID-19 infection rate within different age groups reported in a recent study [39], we identified that the mental stress causes reported in Table 2 also vary with respect to age groups. It is important to note that, in Figure 5, older age groups (60 to 70, 70+) and participants under 20 indicate weak relationships with common causes of mental stress, in contrast to middle-age groups who are grouped together mainly with attributes including pandemic, work, COVID-19, and relationships. This indicates that the middle-age working class population is mentally stressed mainly due to school closure, the pandemic, and relationships, which is not the case with younger and older age groups. However, young participants (20 to 30) indicate substantial correlation with studies and performance that potentially represents students. This also algins with the research outcomes reported in [41] indicating the impact of the effectiveness of vaccination and the pandemic on students’ performance. Likewise, [13,33] reported similar outcomes indicating a negative impact of pandemic over students’ academic performance. Furthermore, perception of vaccination in students has a positive impact on the acceptance of e-learning platforms. Further detailed visualisation for individual age groups is available in Supplementary S1 (Figure S2).

The SOM codes plot for relationships between participants’ age groups and major stress causes is shown in Figure S3 which clearly indicates a minor representation of mental stress factors for younger and older groups that also aligns with the MCA outcomes in Figure 5. In contrast, participants in the age group 31 to 40 indicate more concerns due to study performance and work while middle-age (41 to 60) participants are strongly inter-related with COVID-19, work, school closure and childcare, and the pandemic. These statistical and visual outcomes clearly indicate that there is significant association between varying age groups and factors causing the mental stress.

#### 3.1.3. Ethnicity

One of the unique characteristics of our dataset (compared to most of the literature) is the inclusion of ethnic background. The dataset comprises substantial representations of diverse ethnic groups (mainly white British and Asian/Arabic) listed in Table 2. Figure 5 and Figure S4 (in Supplementary) show the visualisation of first two components (retaining 30% of the variance) for mental stress causes and ethnicity. Distinguishing responses are noticed from different ethnic groups in relation to mental stress causes. For instance, the Asian/Arabic group indicates minor mental stress due to work, pandemic, childcare/school closure as compared to white European and white British population. In contrast, they indicated a partial relationship with vaccine and ‘other’ factors (see dataset attached in Supplementary Material S3).

These outcomes align with the SOM-based visualisations shown in Supplementary S1 (Figures S5 and S6) indicating an independence between Asian/Arabic participants and causes of mental stress due to COVID-19 infection, studies/performance, childcare/school closure, and relationships (*p* > 0.05). In contrast, white British and white EU groups indicated strong associations with these factors. On the other hand, both Asian/Arabic and white British groups indicated vaccination as one of the stress causes, whereas a substantial proportion of the Asian/Arabic population showed finance, vaccination and ‘other’ factors as major causes of stress. These outcomes clearly indicate the significant relationship between ethnic background and factors causing the mental stress.

#### 3.1.4. Profession

Figure S7 (in Supplementary S1) demonstrates 31% of the variance retained in the first two dimensions of MCA. While both groups (i.e., medical and academic professionals) indicated almost similar correspondence to COVID-19 infection, pandemic, and work, people from medical profession indicated comparatively less correspondence to these factors as compared to academic professionals specifically, in the case of studies and childcare/school closure. On the other hand, people in ‘other’ professions indicated high correspondence to ‘other’ stress causes as shown in Table 2. These outcomes align with the SOM code plots shown in Figure S8, indicating interrelationships between profession and stress causes. Additionally, the outcomes endorse findings from existing works [14,35] which reported healthcare workers as being more affected by the COVID-19 and pandemic situation. Similar to MCA, Figure S8 indicates a strong correlation between academic professionals and childcare/school closures as compared to participants from medical and other professions.

While the above MCA- and SOM-based visualisations are useful to understand the diversity of stress causes with respect to sociodemographic factors, we further employed Chi-square test of independence to analyse the statistical significance between these factors and mental stress causes. Table 3 shows the detailed statistical outcomes in relation to investigating the inter-dependence and statistical significance of sociodemographic factors and stress causes. These outcomes further support the investigation of hypothesis (**H_0__1**, **H_1__1**) and, therefore, the argument set in RQ_1_. It can be observed from the statistical outcomes in Table 3 that childcare/school closure indicates a significant dependence with age group and sex with *p* = 2.2 × 10^−16^ ≈ 0 and *p* = 0.02, respectively. This outcome endorses the SOM- and MCA-based visualisations and outcomes (as shown in Figure 3, Figure 4 and Figure 5) indicating females and middle-age populations reporting childcare/school closure as one of the major causes of mental stress due to COVID-19 and its impacts. On the other hand, ethnicity and profession did not indicate significance (*p* > 0.05) which also aligns with the SOM- and MCA-based visual analysis.

In relation to work, pandemic, stress, COVID-19, finance, and studies/performance, several demographic factors indicate significant dependence which also endorses the SOM- and MCA-based outcomes. For instance, profession and age group are significantly dependent with work (age group: *p* ≈ 0, profession: *p* = 0.0003) while sex indicates a significant dependence with the pandemic (*p* = 0.01). Similarly, stress, COVID-19 is significantly dependent with sex (i.e., *p* ≈ 0), profession (*p* = 0.0001), and age group (*p* = 0.01), indicating that mental stress varies with respect to sociodemographic factors that aligns with the MCA-based outcomes in Figure 5, which indicates a discrete-level correspondence between sociodemographic groups and factors causing mental stress.

In summary, the aforementioned statistical outcomes with (*p* < 0.05) and visualisation patterns clearly validate the rejection of **H_0__1** and indicate significant relationships between sociodemographic diversity and several causes of mental stress as listed in Table 3.

### 3.2. Vaccination Acceptability and Perception

Research studies have been reporting varying public perception in relation to COVID-19 vaccination, acceptability and perception. The authors in [42] reported the significant impact of misinformation on vaccination acceptability in the UK and USA. Several studies indicated that children affected by COVID-19 are the potential drivers of COVID-19 spread in the general public [43]. Likewise, the authors in [44] highlighted the risk of increased vulnerability to vaccine-preventable diseases due to a reduction in child vaccinations. However, the acceptability of child vaccination at the current stage is a major challenge. Some recent studies such as [45] highlighted the arguments addressing the advantages and risks for child vaccination. The authors addressed the existing arguments in support of delaying child vaccination as well the opposite, the effectiveness indicated by medical experts regarding child vaccination in previous studies. Likewise, work presented in [46] hypothesised that the measles, mumps, and rubella vaccine is effective for children, however, these studies are not supported by the experimental analysis or public opinions. Alternatively, we believe that the perception about vaccination specifically for the children is highly associated with sociodemographic diversity.

Table 2 and Supplementary S2 (Q13 to Q20) demonstrate the overall distribution of public responses regarding vaccination concerns (e.g., feeling safe if vaccinated) as well as acceptability (e.g., acceptance for family, children, etc.). Around 15.5% of the total participants (i.e., 93 participants) do not feel safe being vaccinated. Most of them (67.3%) show the side effects as a major concern, while 31.7% and 14.4% reported ‘other’ factors and ‘personal beliefs’, respectively, as major concerns regarding the vaccination. Similarly, the acceptability of vaccination varies in public responses. The majority of participants responded with acceptance of vaccination for their family and themselves (80.5% and 70.2%, respectively), while it reduced to 47.2% for the children only. However, a small proportion (32 participants, 5.3%) responded with no acceptance. To investigate the varying distributions of responses in relation to sociodemographic diversity and vaccination perception, following hypotheses are set.

**H_0__2.**  *Sociodemographic diversity is independent to vaccination acceptance in public.*

**H_1__2.**  *Sociodemographic diversity is significantly dependent to vaccination acceptance in public.*

**Figure 6:**
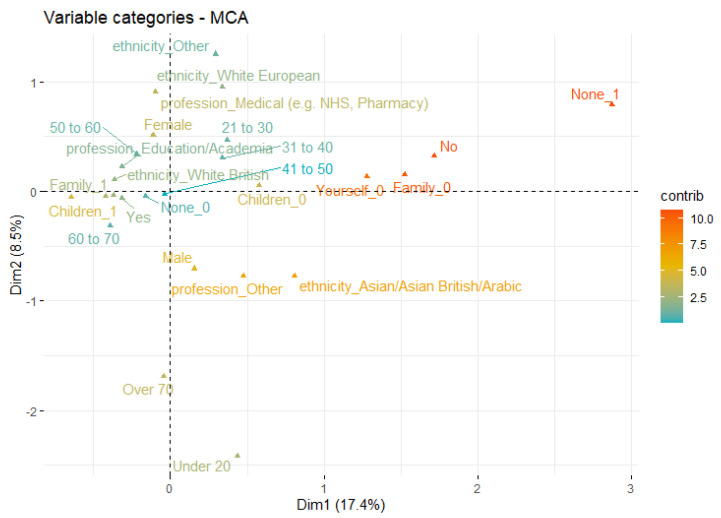
MCA outcomes (first two dimensions) for the combined visualisation of vaccination acceptance and sociodemographic attributes (ethnicity, profession, sex, and age groups).

Similar to mental stress causes, we employed MCA and SOM for the visual analysis of multi-attribute distributions and identification of complex patterns for vaccination perception and acceptance with respect to sociodemographic attributes. Figure 6 summarises the vaccination acceptability and perception within diverse sociodemographic groups represented by two MCA components retaining 26% variance. It can be noticed that participants representing ‘other’ profession and Asian/Arabic backgrounds tend towards less acceptance of being vaccinated as compared to white British from academic and medicine professions. Likewise, males indicate more correspondence to ‘no acceptance’ as compared to female participants.

Figures S9 and S10 (see Supplementary S1) represent the SOM code plots indicating the diversity in public responses about feeling safe if being vaccinated. It is important to note that despite most of the participants (84.5%) feeling safer being vaccinated, *the acceptance rate for the children being vaccinated is comparatively higher in white British than Asian/Arabic and ‘other’ ethnic groups.* Similarly, acceptance to ‘None’ (i.e., complete rejection of being vaccinated) appears mostly for the Asian/Arabic and white European ethnic groups which is not the case for the majority of the white British group.

Likewise, Figures S11 and S12 (see Supplementary S1) show the distributions of vaccination acceptance with respect to profession. It can be observed that responses from ‘other’ professions are highly correlated to ‘None’ (i.e., rejection of being vaccination) as compared to academics and medical professionals. *The acceptance for children is comparatively higher in academic professional than individuals in medical and ‘other’ professions.* Furthermore, vaccination perceived as ‘not safe’ is associated with ‘other’ professionals as compared to people in academia and medical professions.

For the statistical significance, we employed the Chi-square test of independence between sociodemographic factors and vaccination acceptance in public responses. It can be noticed in Table 4 that dependence between ethnicity and vaccination acceptance is significant (*p* < 0.05 in all cases). Similarly, there is a significant dependence between ethnicity and feeling safe (if vaccinated) (*p* = 1.8 × 10^−8^). On the other hand, sex did not indicate significant dependence with vaccine safety (*p* = 0.9) and its acceptability (*p* > 0.05 in all cases). Profession indicates significance for both vaccination safety as well as acceptability (*p* ≈ 0). These outcomes align with the MCA- and SOM-based visualisations in Figure 6 and Supplementary Information S1.

The above findings also endorse similar works presented in previous studies. For instance, [42] presented the impact of online misinformation on vaccination acceptability within the UK and USA. They noticed a substantial decline in acceptance rate due to misinformation, specifically among those who definitely intended to accept the vaccination before such misinformation. Furthermore, the study reported differences in the impact of misinformation on vaccination acceptance between sociodemographic groups. For instance, unemployed individuals in the UK indicated less indecision about vaccination acceptance as compared to employed groups. Likewise, as compared to white ethnicity, ‘other’ ethnic groups and lower-income individuals were more robust to misinformation in the USA. There is also a religious factor indicating differences in opinion between Jewish and Christian participants within the UK. Furthermore, compared to males in the USA, females indicate more likeliness to decrease vaccination acceptance upon exposure to misinformation. A global survey conducted in [47] reported significant differences in vaccination acceptance across the globe, where respondents mainly trust in government information sources about the vaccination. Many recent studies in both the UK and USA have highlighted females as being less likely to get vaccinated than males [20,48,49]. However, our findings indicate an independence between sex and vaccination acceptance. This may be due to several factors such as study design, demographics differences, and mainly time of survey because effective campaigns by policymakers across the globe might have an impact on public perception about COVID-19 vaccination as well as help to counter the misinformation.

In summary, the above statistical results and pattern analysis clearly support the argument that acceptability of vaccination and its perception in public is significantly dependent on several sociodemographic diversity factors (mainly ethnic background, sex, and age Group), and therefore, **H_0__2** can be rejected. The rate of vaccination acceptance across the globe is insufficient, as reported in [47] as well as in the statistical and visual results from the proposed study. These outcomes clearly indicate the need of effective campaigns by policymakers to convince the public to encourage vaccination uptake and compete with misinformation about vaccination.

With the significant contributions and findings, this study has some limitations. For instance, the number of participants (635 participants) could be increased for better generalisation over the population. While the authors made efforts towards a balanced representation from different groups, we could also utilise post-stratification weighting to balance the sample with respect to sociodemographic representations. However, previous research from [12] reported no significant difference in the outcomes from an unweighted sample and balanced representation using the post-stratification approach. Despite the dataset being acquired from diverse ethnic groups, additional geographic locations could produce better representation. Finally, the self-reported responses to survey questions may also be biased and may differ from clinical diagnosis.

## 4. Conclusions

The proposed study investigates the major factors causing the mental stress during and post-COVID-19 vaccination and pandemics within the diverse sociodemographic groups in the UK. While there exist various studies addressing the mental health issues in the related topic, these works are lacking in multiple aspects as compared to the proposed study. For instance, most of the works are based on surveys, however, the datasets are not publicly available, which could be very useful and informative for the corresponding authorities, the public, and academic stakeholders. Secondly, there is very limited diversity in the existing related works mainly in relation to ethnicity and professional backgrounds of the surveyed population. Likewise, in contrast to our work, the existing survey designs lack information requests from participants about how they coped with challenging situations, such as the pandemic. This might be useful for the future epidemics as well as longer-term mental health issues. Finally, and most importantly, most of the literature uses conventional statistical approaches that are impractical to effectively visualise the complex patterns and associations within the multi-dimensional data. In contrast, we performed detailed analysis using advanced statistical tools and an intelligent pattern-matching algorithm over a primary dataset comprising participants from different professions, age groups, ethnic backgrounds, and sexes. Our outcomes indicated significant relationships between sociodemographic factors and major stress causes, mainly the pandemic, work, COVID-19, school closure, and social relationships. Interestingly, majority of the participants (40.3%) speak to family or friends and watch TV shows (34%) to reduce the mental stress which also endorsed the outcomes reported in a previous study [14]. We furthermore observed substantial differences about vaccination perception and its acceptance within different sociodemographic groups which might be helpful for understanding the variations in diverse communities. As an example, the vaccination acceptance for the children is comparatively higher in white British than Asian/Arabic and ‘other’ ethnic groups. Similarly, acceptance to ‘None’ (i.e., complete rejection of being vaccinated) is higher in Asian/Arabic and white European ethnic groups which is not the case for the majority of the white British group. Furthermore, vaccination being felt as ‘not safe’ is significantly associated with ‘other’ professionals as compared to academic and medical professionals. Despite a substantial proportion of the participants (84.5%) feeling that COVID-19 vaccination is ‘safe’, *the acceptance rate for the children being vaccinated is far lower (only 47%) which might be a concern in various parts of the globe. The acceptance for children is comparatively higher in academic professionals than individuals in medical and ‘other’ professions.* This clearly indicates the need of potential vaccination campaigns for the target audience.

While the majority (around 58%) of the participants feel a reduction in mental stress level after vaccination, it is not the case for rest of the 42%, which is a considerable proportion. In addition, the primary dataset presented in this study includes additional information related to other social factors such as online shopping, etc. (see Supplementary S1 for detailed survey questions and responses). The outcomes from this study warrant the consideration of mental health interventions during and beyond the pandemic to address the impacts of the COVID-19-related challenges for future pandemics. The study outcomes along with the primary dataset might be useful to help us be better-prepared for future epidemics, as well as in policymaking, mainly related to short- and long-term mental health issues within the various sociodemographic groups. Possible future works may include collecting larger sample across the globe (for better generalisation) and exploiting the mass of available information (from existing COVID-19 and other infectious diseases) as the baseline to train the machine intelligence models (e.g., reinforcement learning, deep transfer learning) for the autonomous and early interventions to combat the challenges associated with infectious diseases.

## Figures and Tables

**Table 1:** Summary of recent works addressing the mental health problems associated with the COVID-19, pandemic and other related challenges.

Study/Method	Outcomes	Public Data	Comments
Ref. [15] Analysis of psychological stress associated with COVID-19 vaccination in China. Multi-linear regression (MLR) is used to identify the strength of the relationship	Higher stress level is noticed in participants with a low level of education, young age, history of chronic disease, mistrust of the vaccination	No	It would be better to ask participants about how they cope with mental stress. No multi-dimensional pattern analysis is performed
Ref. [12] 1024 parents were surveyed to analyse the parental stress and occurrence of adverse childhood experience (ACE) during the pandemic in Germany	Over 50% of the parents reported being stressed by social distancing and the closure of schools and childcare facilities. Up to 33% of the sample reported ACEs in the child’s lifetime	No	Simple frequency and correlation statistics are measured indicating the association between individual factors. No multi-dimensional pattern analysis is performed
Ref. [13] Gender differences in the mental health of university students during the COVID-19 pandemic is addressed using an online survey of 366 undergraduates	Female students are more affected by isolation in the pandemic as compared to males. Regardless of gender, the pandemic has a negative impact on social relationships	No	Study reported the use of social media as a coping strategy with the pandemic situation, however, it affected the students’ academic performance. No multi-dimensional pattern analysis is performed
Ref. [33] Stress, anxiety and depression among undergraduate students during the pandemic is addressed. Responses from 2059 students are considered for the analysis using MLR	Females, rural, low-income, and academically underperforming students are identified as being more vulnerable to mental stress, anxiety and depression	No	Study mainly focuses on pandemic-related issues. The authors could also include a survey question about coping strategies for these challenges. No multi-dimensional pattern analysis is performed
Ref. [14] Anxiety, stress, depression, and fear during the pandemic in healthcare workers (365 survey participants) are analysed in Jordan	Psychological distress is associated with multiple factors including being male, married, aged 40 years and older, and having more clinical experience. Study also reported that social support during the pandemic is primarily dependent on support from their families, followed by support from friends	Yes	Authors may use appropriate data transformation (e.g., one-hot encoding). Study also addressed some strategies to mental health issues (e.g., social support during pandemic). Sample is comparatively small. No multi-dimensional pattern analysis is performed
Ref. [34] Pandemic impact on the mental health of adults in Bangladesh is addressed. A total of 1427 participants were surveyed and analysed using MLR.	Study reported a high level of: -Anxiety within the older age group (40 plus), females, low education level, and housewives -Depressive symptoms in youth (23 years and younger), females, and unemployed people -Stress in females and unemployed people.	No	Study addresses the sociodemographic factors, including gender, age, educational level, religion, etc., however, with limited diversity (e.g., one ethnic background and 75% male participants). No multi-dimensional pattern analysis is performed
Refs. [35,36,37] Review studies on mental health issues associated with COVID-19, vaccination, and the pandemic	[37] COVID-19 has direct and indirect impacts on mental health. [35] Healthcare workers, children, students, people with existing psychiatric disorder, and adolescents have been particularly affected by the pandemic. [36] Depression, anxiety, and psychological distress are highly associated with the pandemic. There is no significant difference in gender and geographic locations	NA	Recommended non-psychiatrist coping mechanisms to help prevent mental health issues. Large subjective variations exists in relation to mental health issues, indicating the need to provide personalised help and interventions (for post-pandemic and future epidemics) mainly to the vulnerable groups.

**Table 2:** Distribution of public responses (i.e., attributes) to outlined survey questions. Detailed visualisations of attribute distributions are presented in Supplementary S2.

Attribute	n(%)	Attribute	n(%)	Attribute	n(%)
Ethnicity					
- Asian/Arabic	146(24)	Profession		age-Group	
- White British	386(64)	- Education	284(47)	- Under 20	12(2)
- White EU	51(8)	- Medical	107(18)	- 21–30	68(11)
- Other	17(3)	- Other	209(35)	- 31–40	113(19)
		Stress reduced		- 41–50	156(26)
Safe vaccine		- yes	283(47)	- 51–60	137(23)
- yes	507(84)	- no	198(33)	- 61–70	81(14)
- no	93(16)	- do not know	119(20)	- Over 70	33(6)
				Stress-causes	
				□ Work	203(48)
Sex		Stress COVID		□ Pandemic	183(43)
- male	252(42)	- yes	402(67)	□ COVID-19 (infection)	152(36)
- female	348(58)	- no	198(33)	□ Childcare/School	105(25)
Type of vaccine				□ Relationships	92(22)
- None	123(20)			□ Other	54(12)
- pFizer-BioNtech	180(30)			□ Finance	46(11)
- Oxford-AstraZeneca	290(48)			□ Studies/perf	42(10)
- Other	7(1)			□ Vaccine	39(9)
Side effects of vaccine					
□ Tired	228(47)			Shopping online	
□ Muscle-Pain/Swell	184(38)	Stress management		- yes	396(66)
□ Headache	175(36)	□ Speak to family	242(40)	- no	204(24)
□ Chill/Aches	166(34)	□ Watch TV, etc.	203(34)	Social media time	
□ Fever	106(22)	□ Engage in hobbies	178(30)	- yes	577(96)
□ None	95(20)	□ No stress	123(20)	- no	23(4)
□ Strange feeling	52(11)	□ Sports/games	111(18)	Stress-shopping	
□ Nausea	48(10)	□ Other	83(14)	- yes	350(58)
□ Dizziness	36(8)	□ Social media use	79(13)	- no	250(42)
Accept vaccine		Concerns vaccine	93 (15)	Future SOP	
□ Yourself	451(75)	□ Side effects	70(67)	□ Mask wear	328(55)
□ Children	283(47)	□ Other	33(32)	□ Social distance	291(48)
□ Family	483(80)	□ Personal beliefs	15(14)	□ Tier response	197(33)
□ None	32(5)	□ Allergic	9(8)	□ No restriction	167(28)
□ Do not know	37(6)	□ Needle-phobia	4(4)	□ Lockdown	81(13)

**Table 3:** Statistical significance of relationships between major causes of mental stress reported in the dataset and sociodemographic diversity.

	Childcare and School Closure			Work			Vaccine			Pandemic		
	X^2^	Df	*p*-Value	X^2^	df	*p*-Value	X^2^	Df	*p*-Value	X^2^	df	*p*-Value
Ethnicity	4.9	3	0.11	5.8	3	0.12	19	3	0.0002	4.5	3	0.21
Age group	88	6	2.2 × 10^−16^	38	6	9.4 × 10^−7^	11	6	0.08	12	6	0.06
Sex	5.3	1	0.02	1.8	1	0.17	0	1	1	5.7	1	0.01
Profession	3.6	2	0.12	15.6	2	0.0003	2.3	2	0.3	1.9	2	0.3
	**Relationships**			**Stress_COVID**			**Finance**			**Studies/Performance**		
Ethnicity	6.9	3	0.07	6.9	3	0.07	18.4	3	0.0003	21	3	0.00001
Age group	10.4	6	0.1	16	6	0.01	11.7	6	0.06	45	6	4.2 × 10^−8^
Gender	1.9	1	0.15	21.4	1	3.5 × 10^−6^	0	1	1	0.8	1	0.3
Profession	0.6	2	0.7	17.6	2	0.0001	7.5	2	0.02	2.6	2	0.2

**Table 4:** Statistical significance of relationship between sociodemographic attributes, vaccination acceptability, and its perception.

	Safe Vaccine		Vaccine Acceptance							
			Yourself		Children		Family		None	
	X^2^	*p*	X^2^	*p*	X^2^	*P*	X^2^	*p*	X^2^	*p*
Age group	22	0.0009	10	0.1	23	0.0005	7	0.34	9	0.16
Sex	0.01	0.91	13	0.7	0.8	0.35	3.8	0.05	0.1	0.7
Profession	12	0.002	20	4 × 10^−5^	20	3.7 × 10^−5^	19	6.3 × 10^−5^	9	0.01
Ethnicity	38	1 × 10^−8^	48	2 × 10^−10^	35	1.1 × 10^−7^	31	7.8 × 10^−7^	17	0.0007

## Data Availability

The data presented in this study are available in [Supplementary S3].

## Supplementary Materials

The following supporting information can be downloaded at: https://www.mdpi.com/article/10.3390/ijerph191912932/s1, Supplementary S1: This file contains the additional visualization results in support of Results Section of main manuscript. Supplementary S2: This file contains the Questions asked in the survey and the distribution of public responses for the corresponding questions. Supplementary S3: This file contains the dataset which represents the pubic responses per each survey question.
