# Supplementary material for: COVID-19 Vaccination and Mental Stress within Diverse Sociodemographic Groups
Source: Int J Environ Res Public Health. 2022 Oct 9;19(19):12932. doi: 10.3390/ijerph191912932 (PMC9565099; doi:10.3390/ijerph191912932)
Supplement: Supplementary file 1 [file ijerph-19-12932-s001.zip › Supplimentary Information S1.pdf]

Supplementary Information S1

This file contains the additional visualization results in support of Results Section of main manuscript.

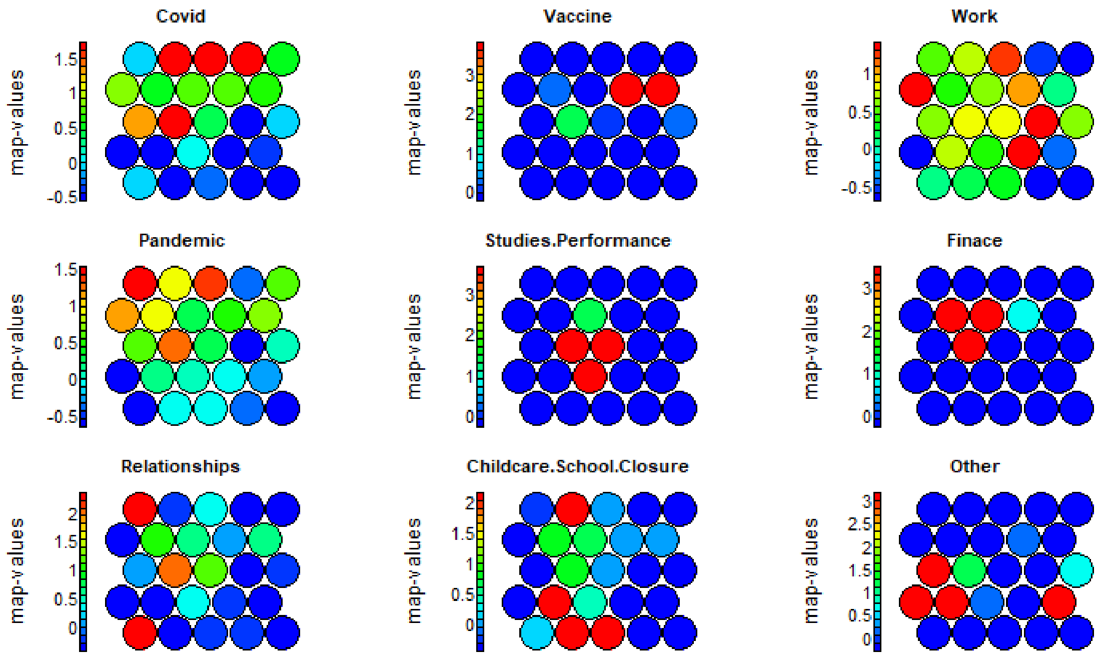

Figure S1.S1. SOM heatmap for inter-relationships between major stress factors. The plot can also be compared with the gender code plot (Figure 2 in manuscript)

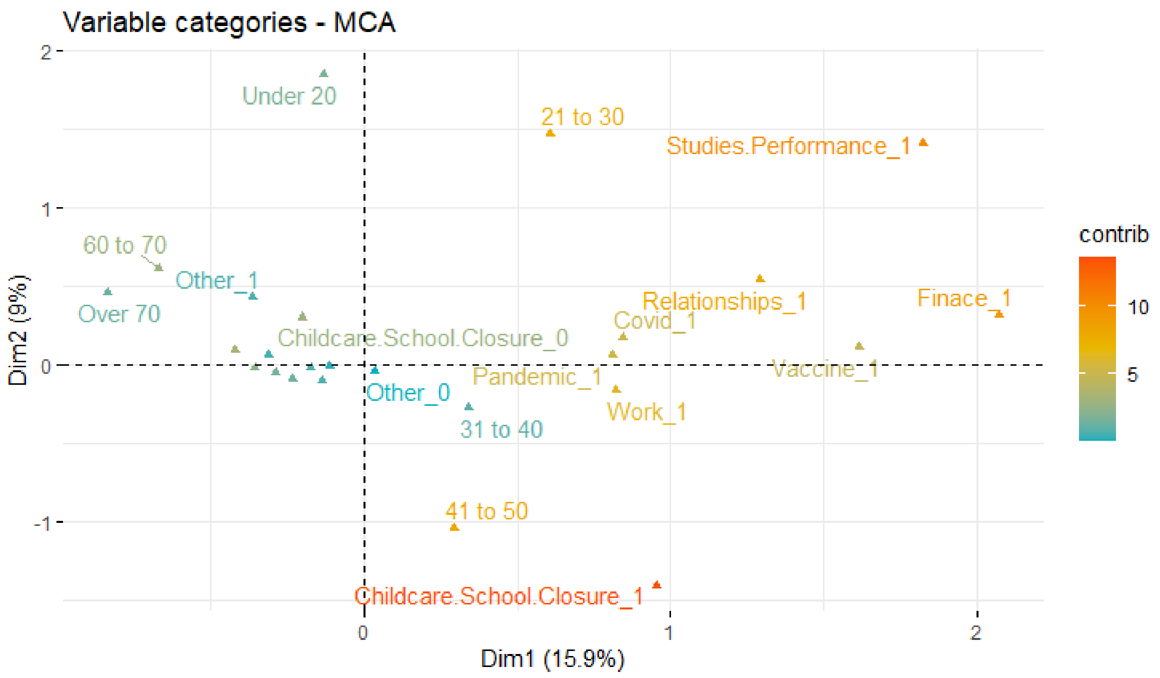

Figure S2.S1. MCA outcomes (1st two dimensions) indicating age-group correspondence to major stress causes reported in public responses. Closer the attributes, higher the correspondence and vice versa. Green to red 'contrib' colour scale indicates the contribution level being low to high respectively for corresponding dimension of MCA

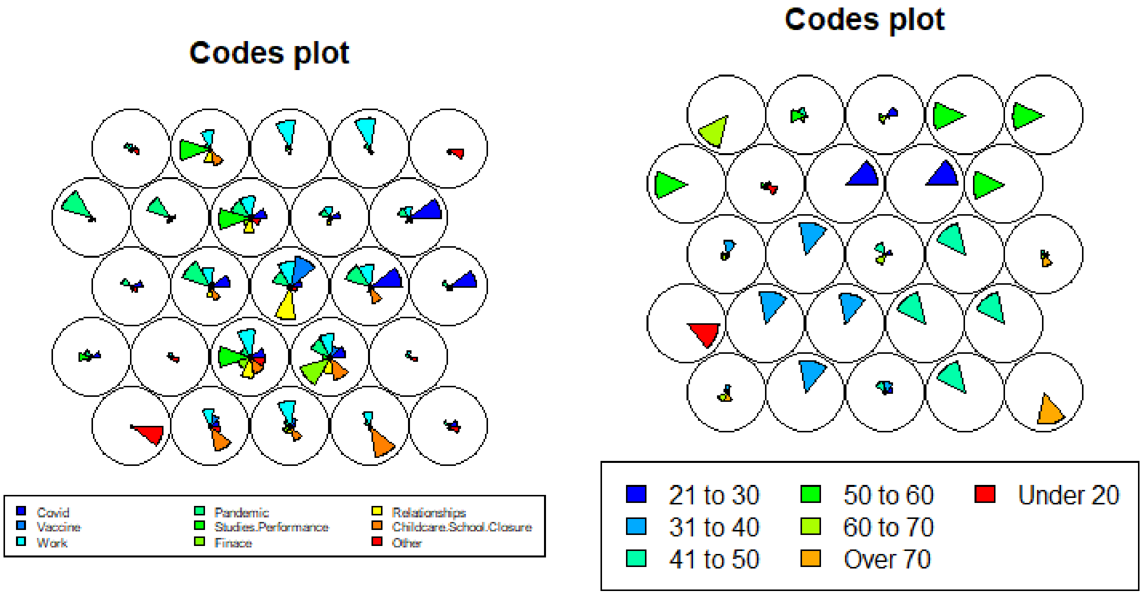

Figure S3.S1. SOM code plot for two-dimensional visualisation of inter-relationships between multiple stress causes (left side plot) within the dataset and participants' age group.

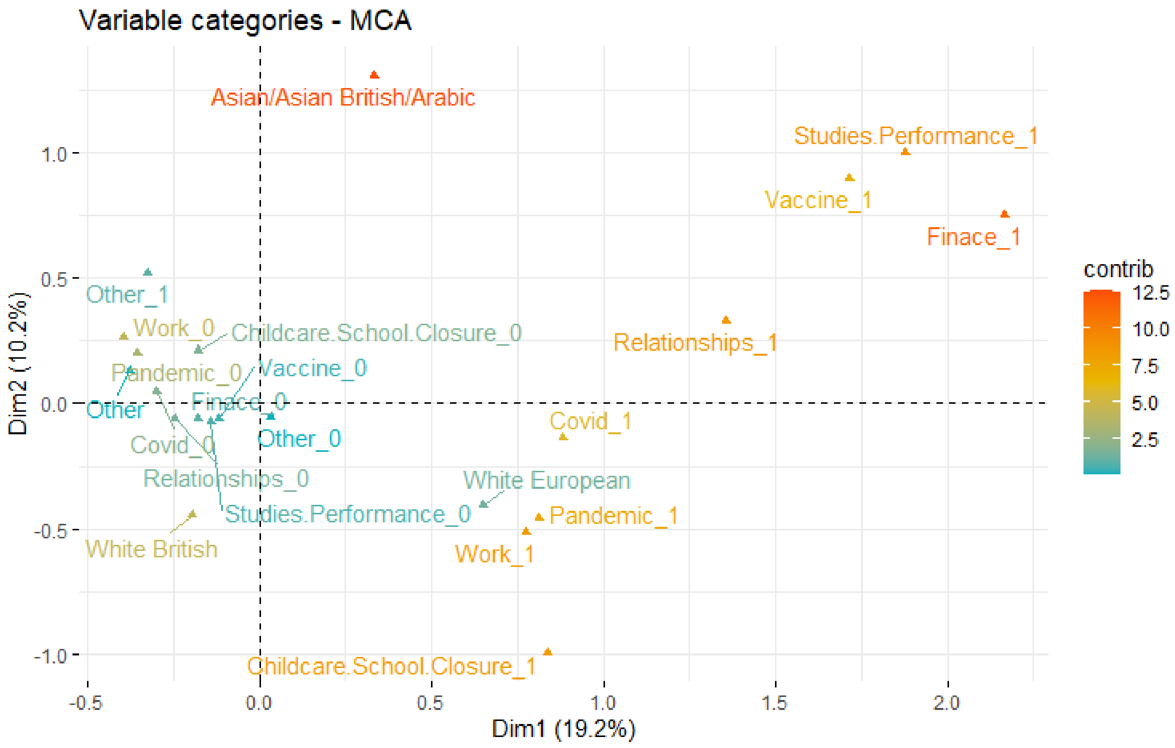

Figure S4.S1. MCA outcomes (1st two dimensions) indicating ethnicity correspondence to major stress causes reported in public responses

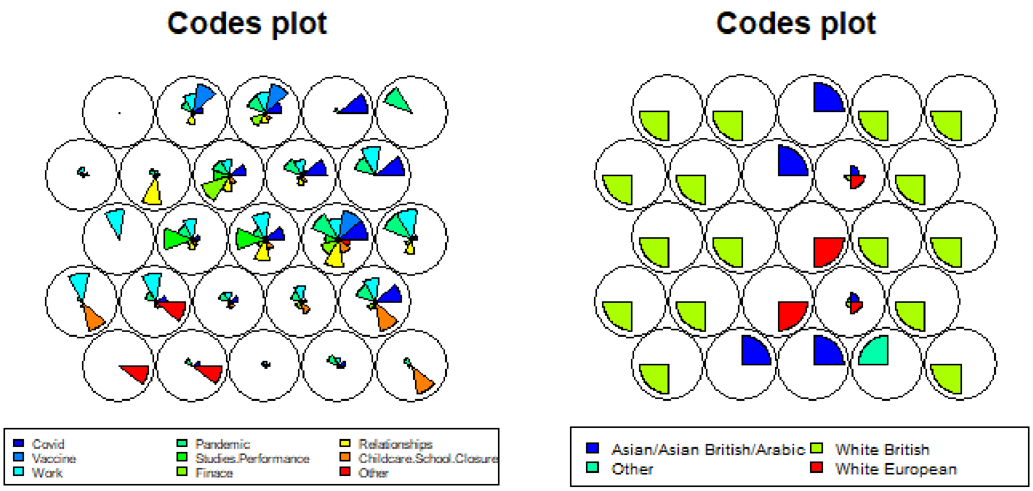

Figure S5.S1. SOM code plot for two-dimensional visualisation of inter-relationships between multiple stress causes (left side plot) within the dataset and participants' ethnicity

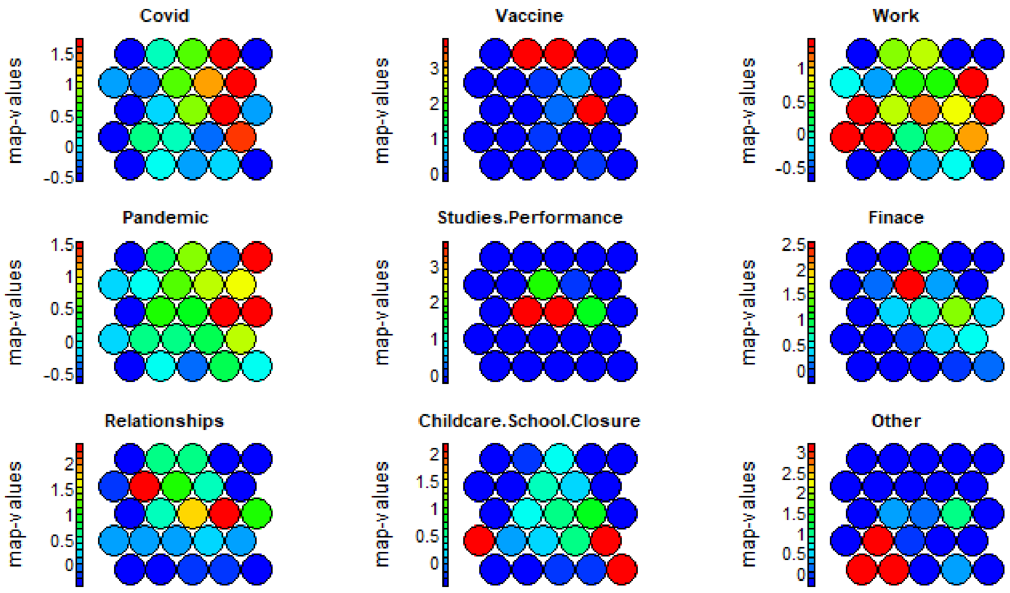

Figure S6.S1. SOM heatmap for inter-relationship between major stress factors and ethnicity (see code plot in Fig.5.S1 above)

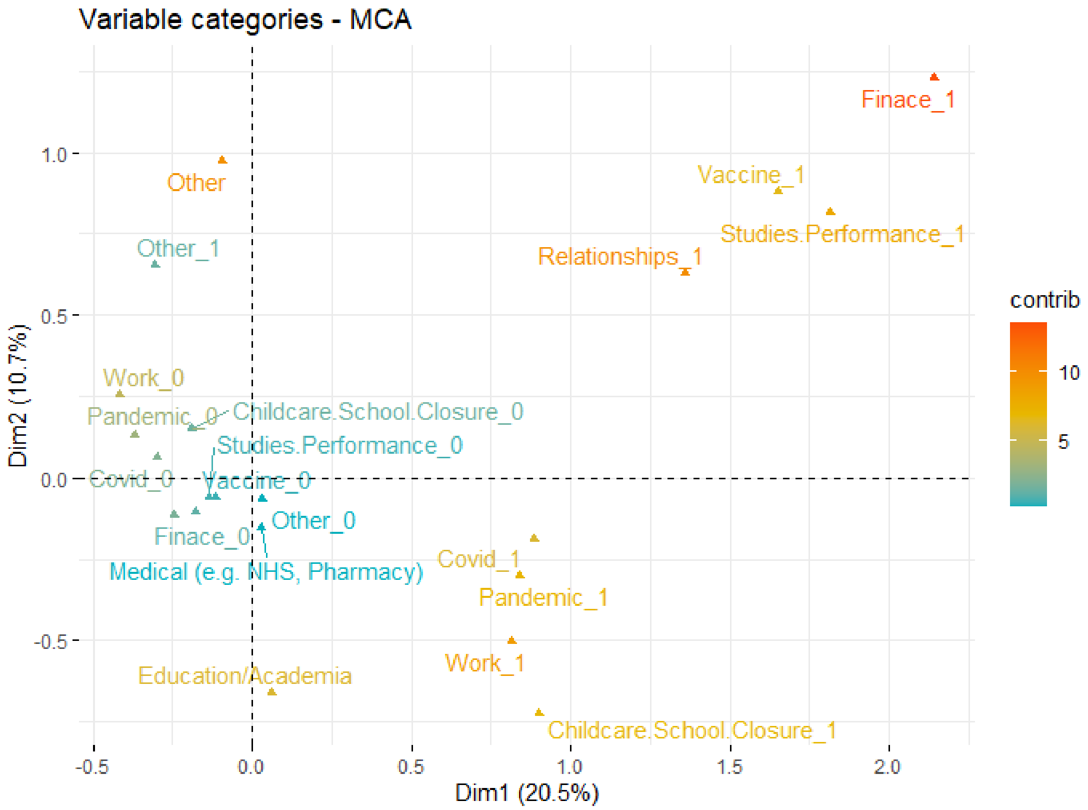

Figure S7.S1. MCA outcomes (1st two dimensions) indicating profession correspondence to major stress causes reported in public responses

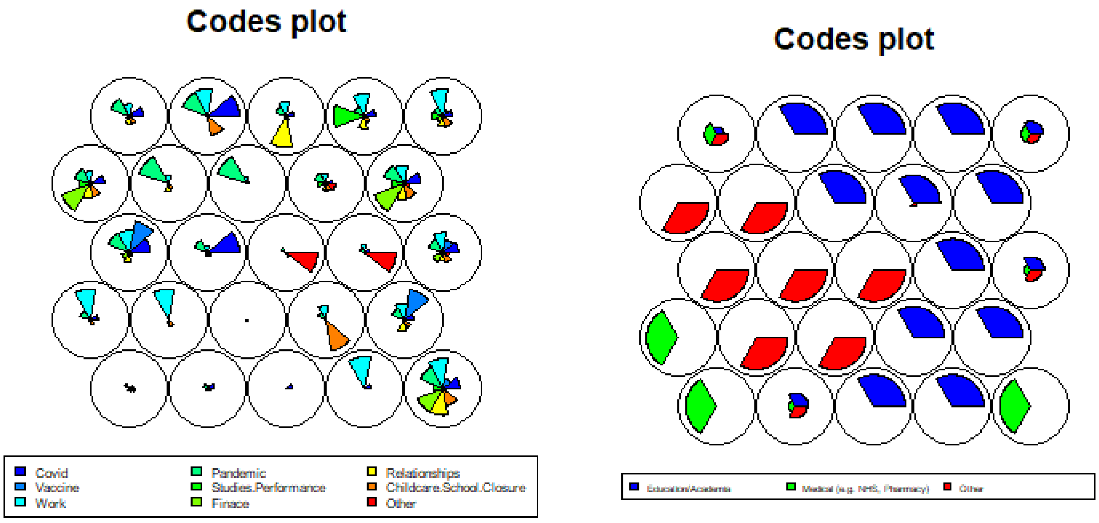

Figure S8.S1. SOM code plot for two-dimensional visualisation of inter-relationships between multiple stress causes (left side plot) within the dataset and participants' profession

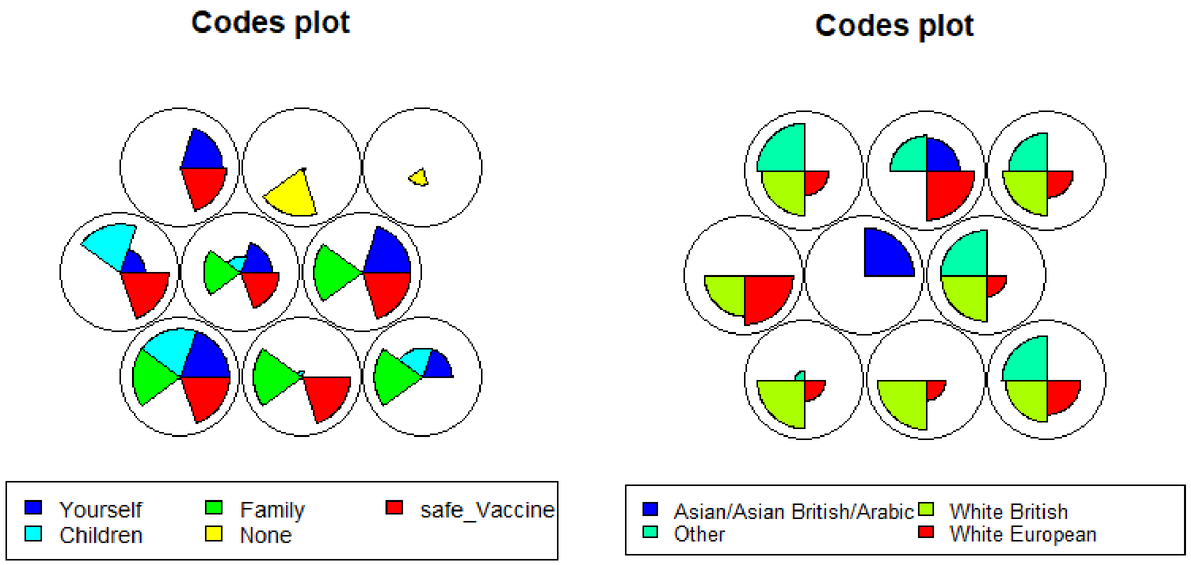

Figure S9.S1. SOM code plot for two-dimensional visualisation of inter-relationships between vaccination acceptance (left side plot) within the dataset and participants' ethnicity

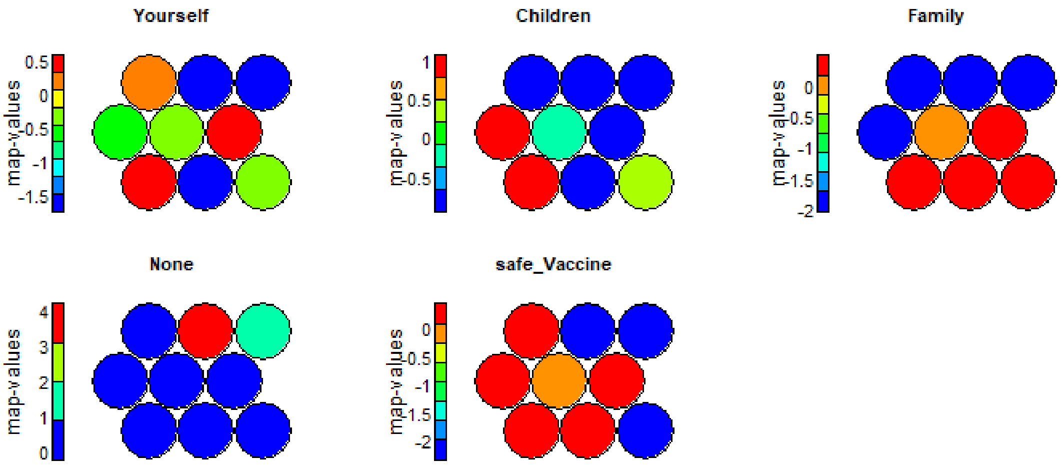

Figure S10.S1. SOM heatmap for individual relationships of vaccination acceptance and participant's ethnicity (see code plot in Fig.9.S1 above)

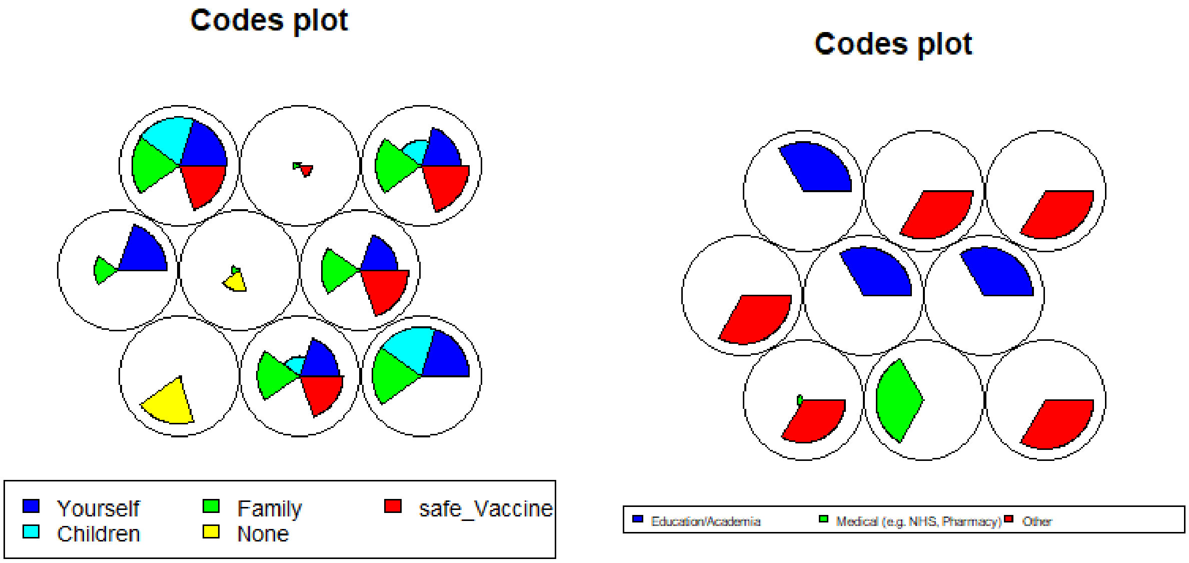

Figure S11.S1. SOM code plot for two-dimensional visualisation of inter-relationships between vaccination acceptance (left side plot) within the dataset and participants' profession (right side plot)

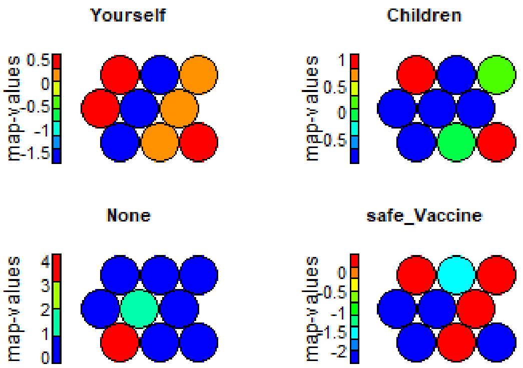

Figure S12.S1. SOM heatmap for individual relationships of vaccination acceptance and participant's profession (see code plot in Fig.11.S1 above)

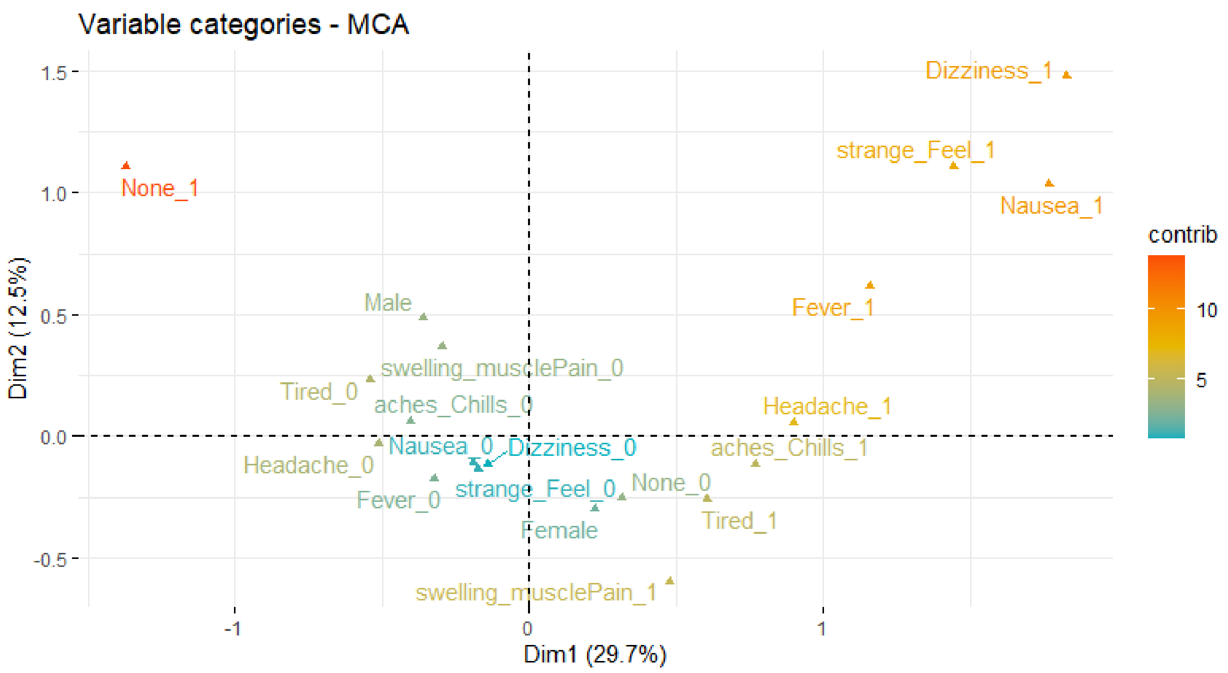

Figure S13.S1. MCA outcomes (1st two dimensions) indicating participants' gender correspondence to vaccination side effects

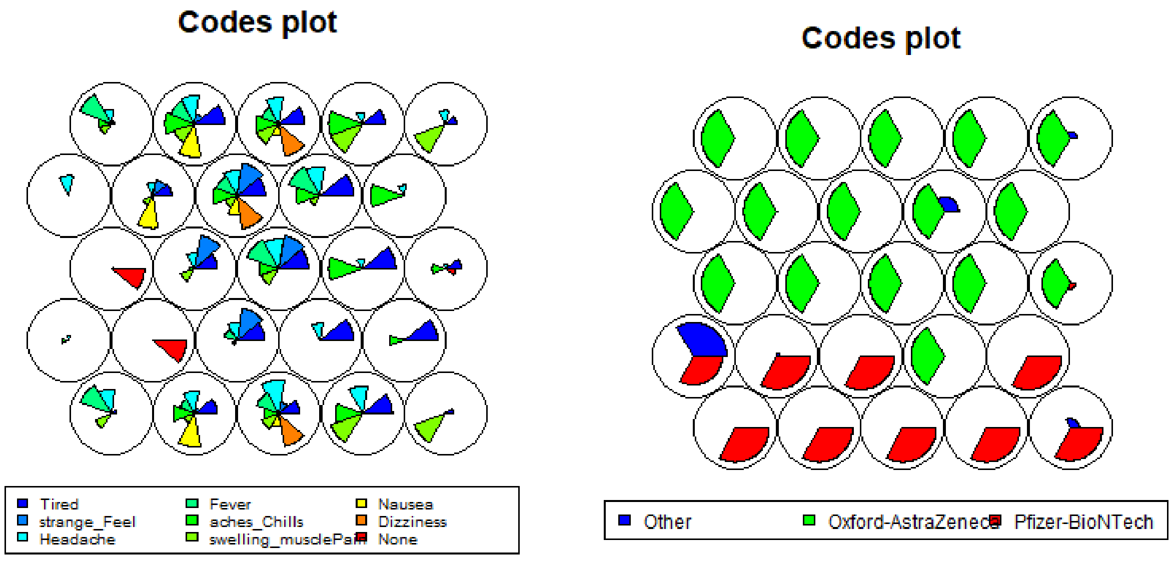

Figure S14.S1. SOM code plot for two-dimensional visualisation of inter-relationships between vaccination side effects (left side plot) and vaccination type (right side plot)
