# Supplementary material for: COVID-19 Vaccination and Mental Stress within Diverse Sociodemographic Groups
Source: Int J Environ Res Public Health. 2022 Oct 9;19(19):12932. doi: 10.3390/ijerph191912932 (PMC9565099; doi:10.3390/ijerph191912932)
Supplement: Supplementary file 1 [file ijerph-19-12932-s001.zip › Survey Outcomes.pdf]

Supplementary Material (S2)

<u>This file contains the Questions asked in the survey and the distribution of public responses for the corresponding questions.</u>

Q1. What is your Age group?

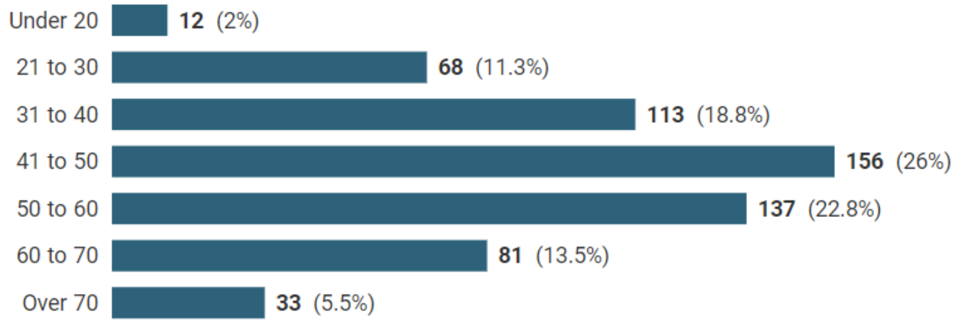

Q2. What is your Gender?

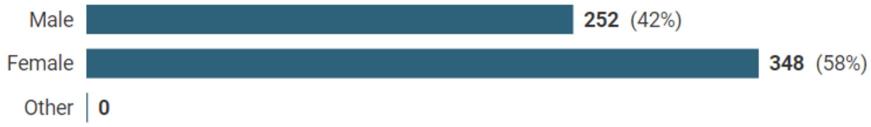

Q3. What is your Ethnic background?

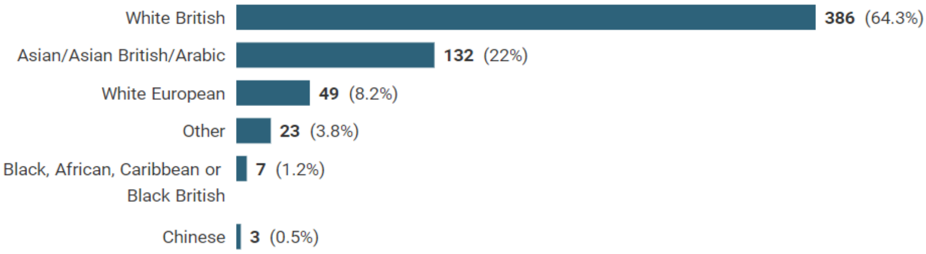

Q4. Which Professional sector do you belong to?

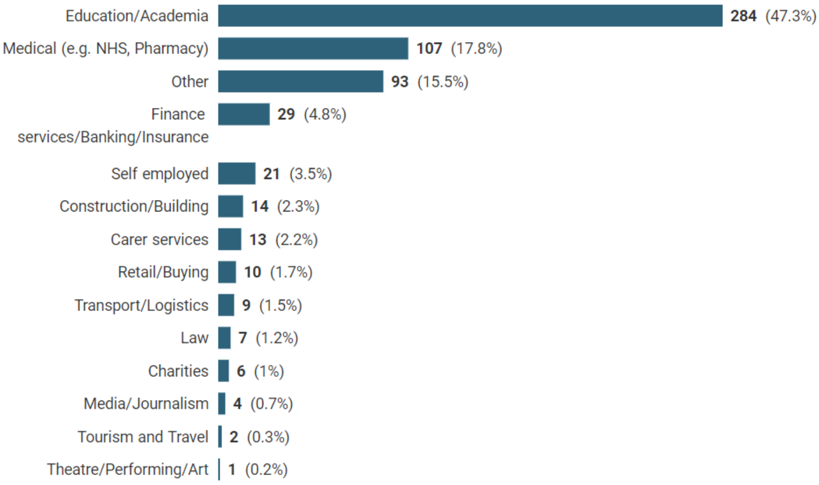

Q5. Do you have any of the below diseases/illness?

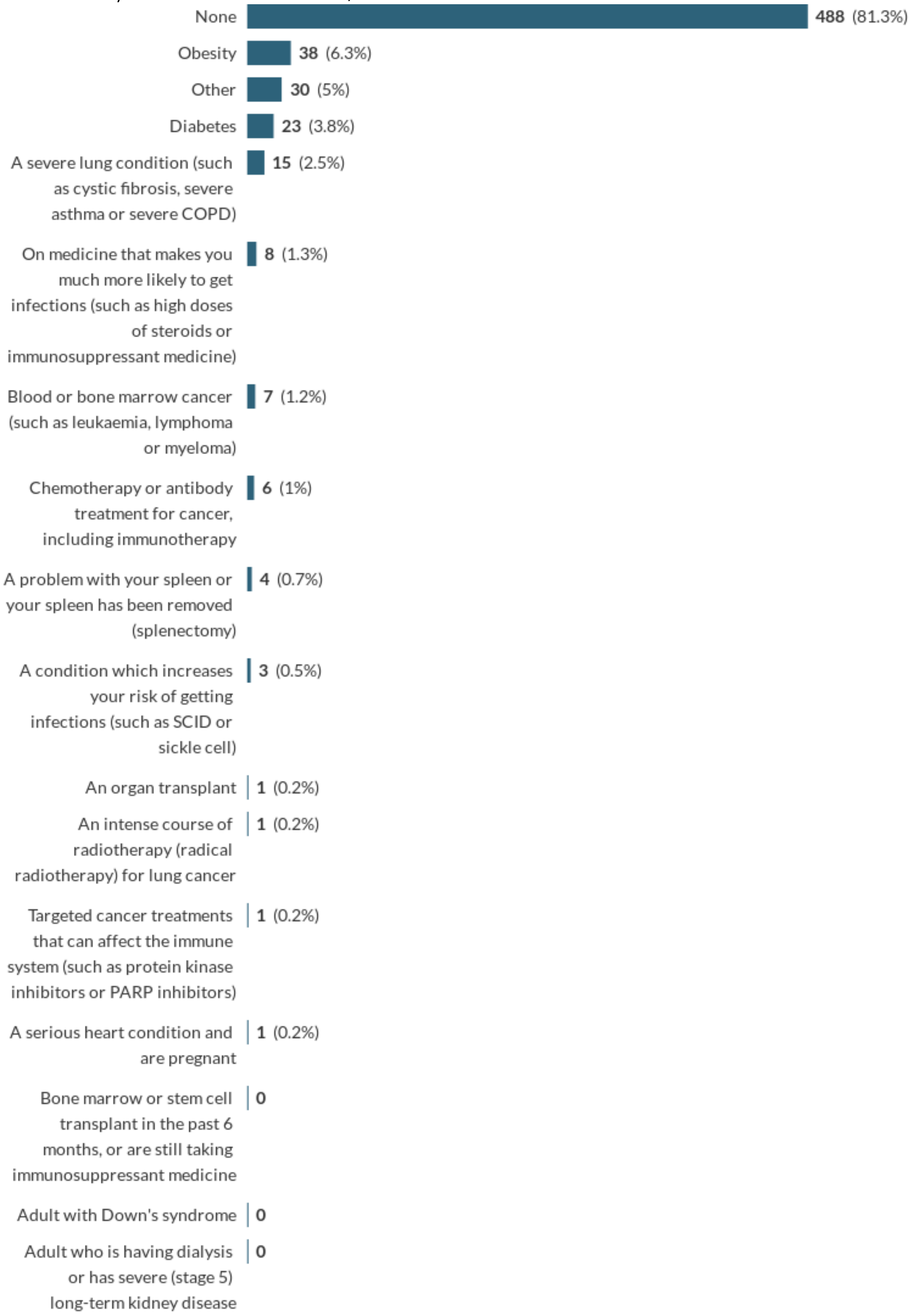

Q6. When did you test positive for Covid-19?

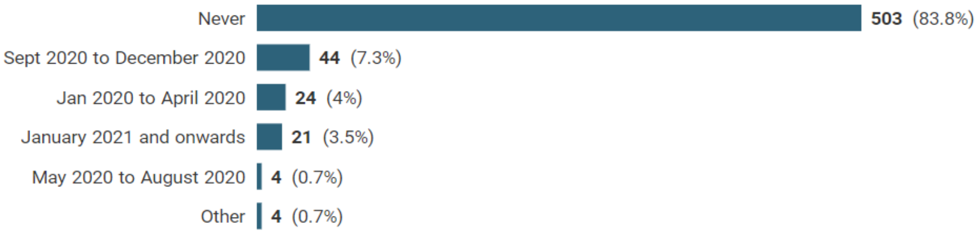

Q7. What was the severity of your symptoms over COVID-19?

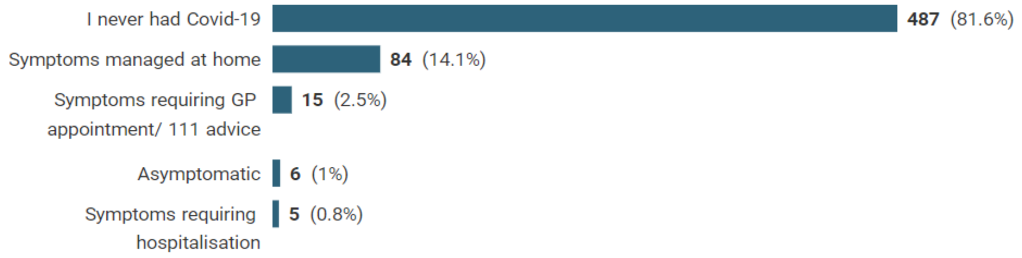

Q8. How long were you actually ill?

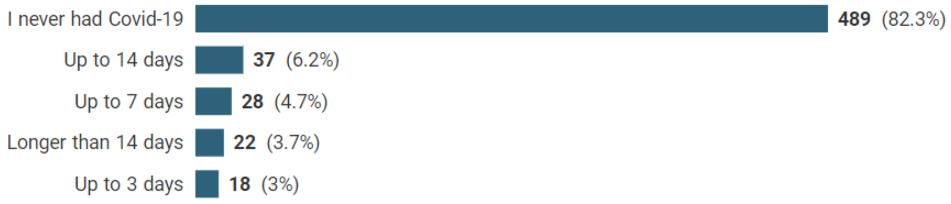

Q9. Do you believe you suffer from 'Long COVID'?

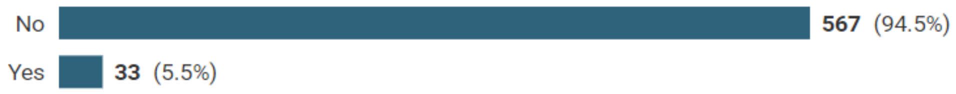

Q9.a. If yes, have you been referred for management?

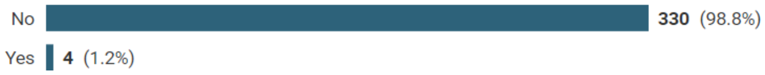

Q10. What was the effects on your personal life? Tick all that apply

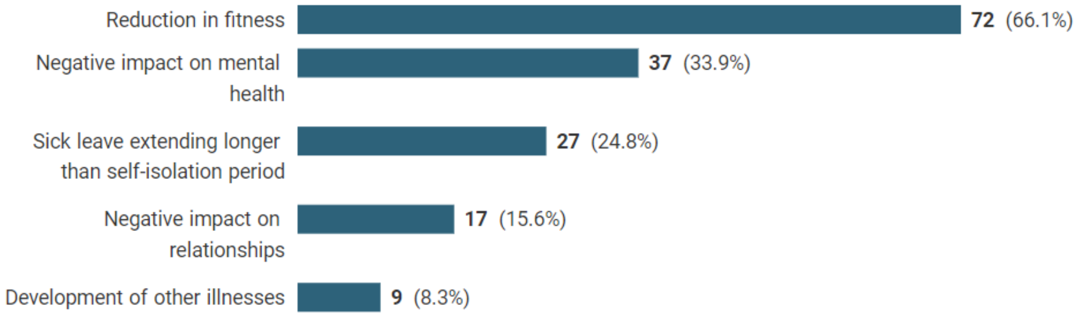

Q11. Did you ever feel Stress due to Covid-19 or its effects on your life?

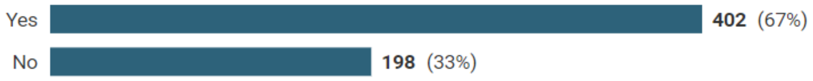

Q11.a. If **yes**, then what was the major **cause** of Stress?

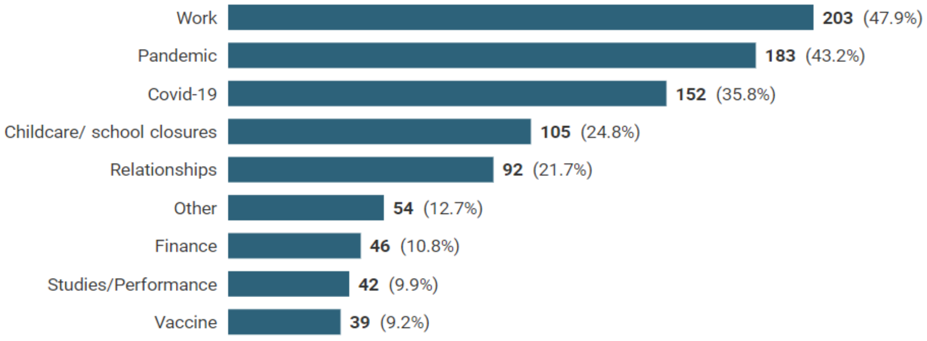

Q12. And what you do if feel stressed (to reduce your stress level)?

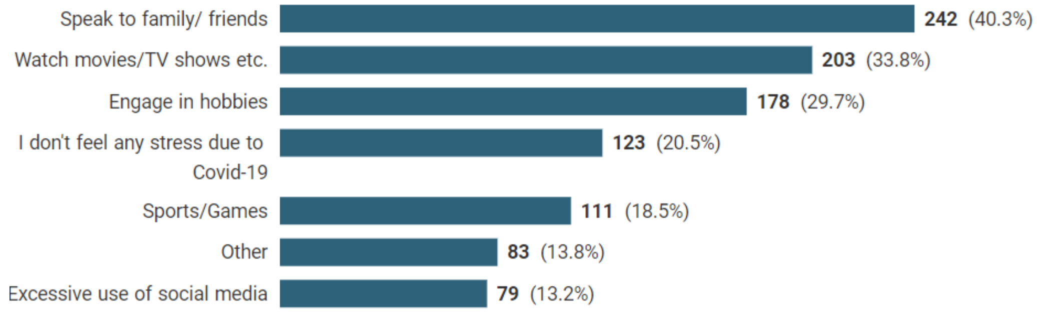

Q13. Are you aware of Covid-19 Vaccine?

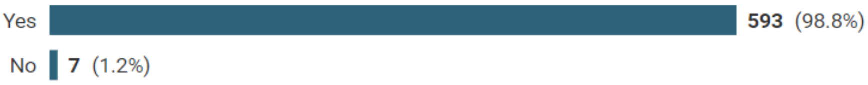

Q14. Which type of Covid-19 vaccine did you take (if any)?

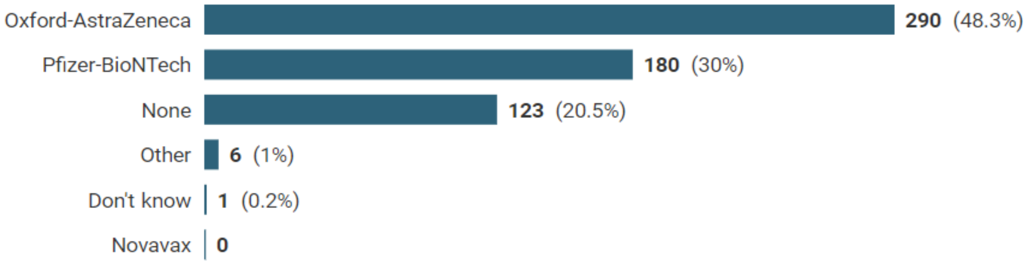

Q15. Do you feel less stress after the vaccination?

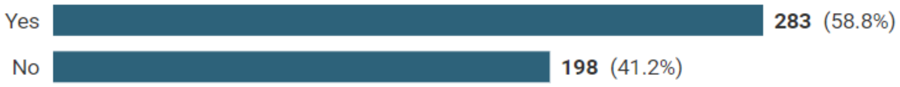

Q16. Which of the following side effects did you feel due to Covid-19 vaccination?

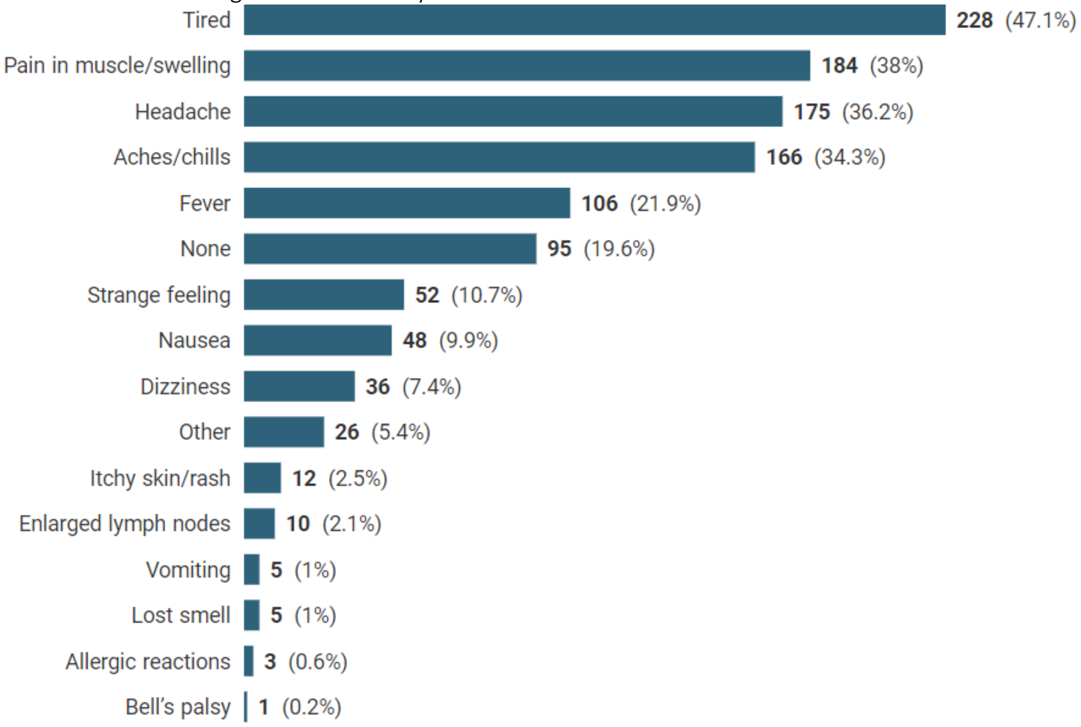

Q17. What was the duration of after-affects?

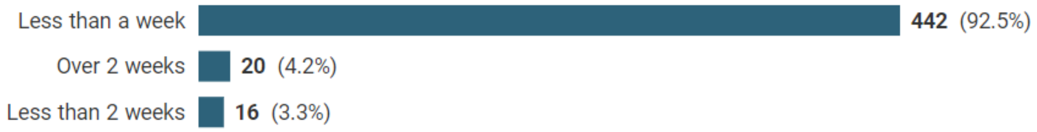

Q18. Would you be happy for the vaccine to be given to:

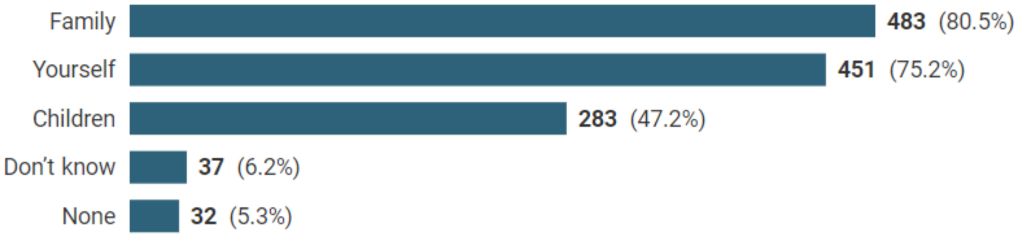

Q19. Do you feel safe having the vaccine?

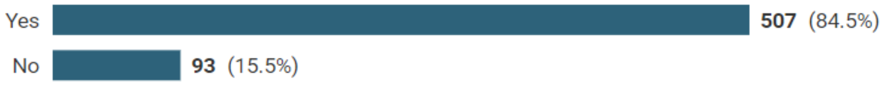

If No, what is your concern?

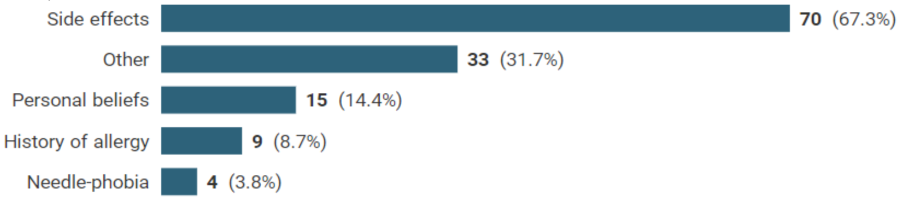

Q20. Following complete vaccination of the population, please select what measures you feel are appropriate:

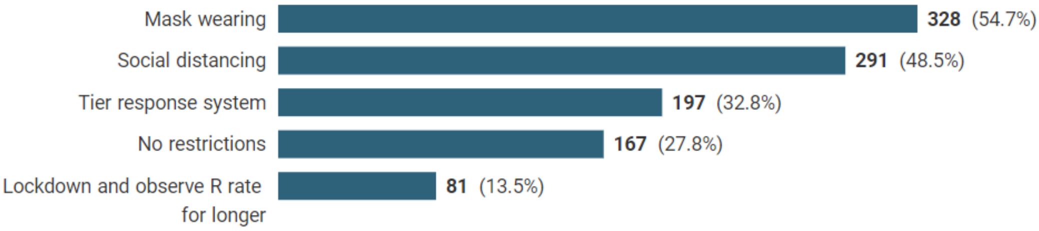

Q21. Is your time spent on TV/Entertainments/Social Media Increased during pandemic?

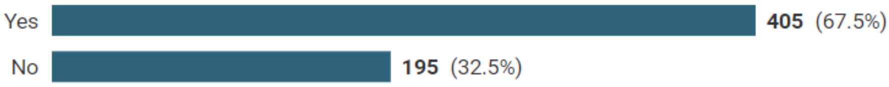

Q22. Do you try your best to have social distancing?

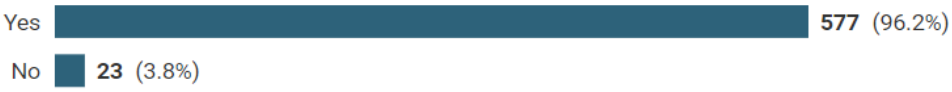

Q23. Do you feel that shopping is stressful for the current situation?

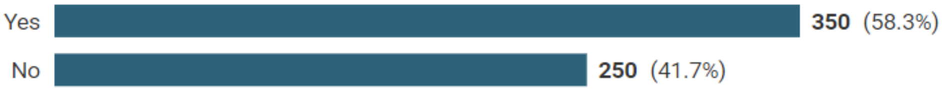

Q24. Do you do online shopping due to Pandemic?

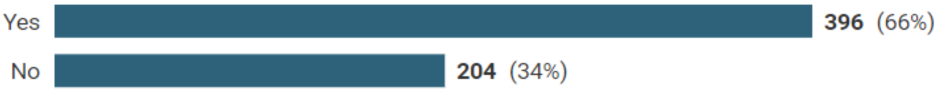

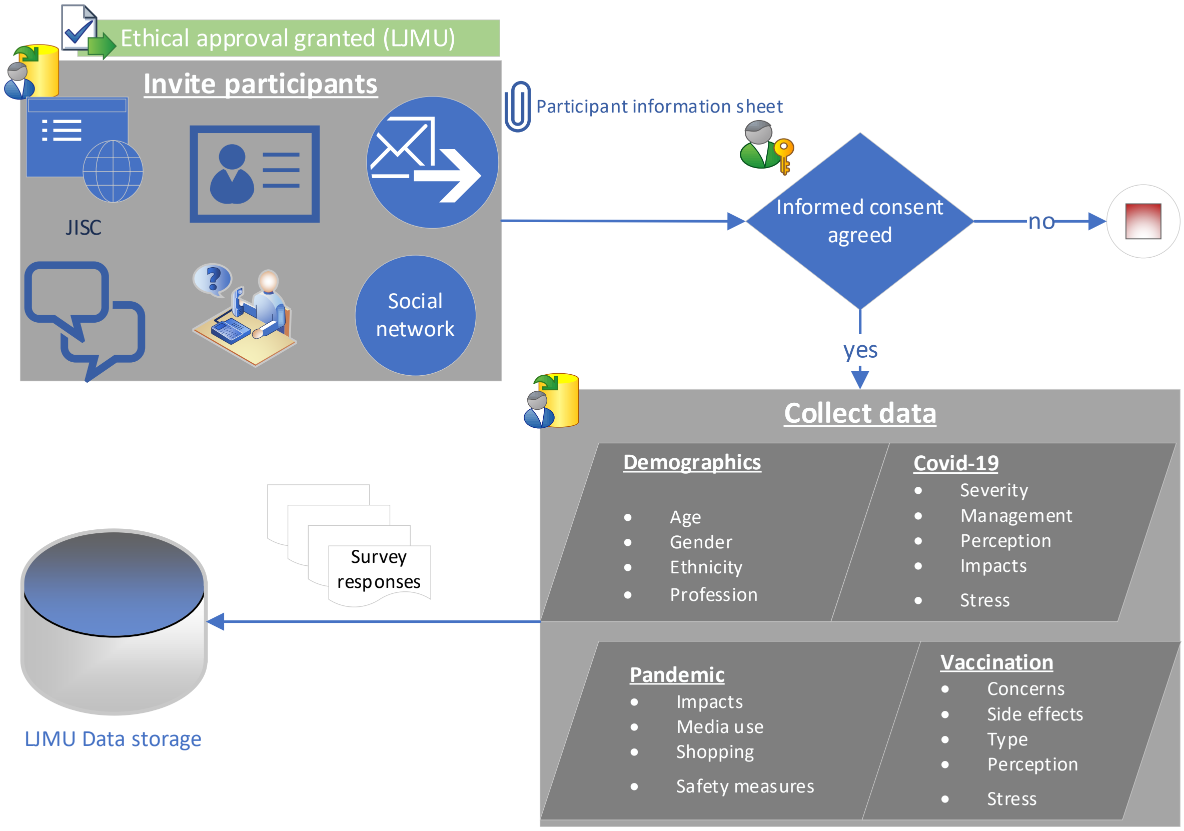

Figure 1.S2. Sequential procedure for primary data collection in proposed study. Following the ethical approval, participants were invited via social and academic networks. All participants were agreed to informed consent before proceeding to survey questions. Online link in the 3rd step was provided to participants and their responses were collected w.r.t demographics, Covid-19, Pandemic, and Vaccination. Finally, the survey data is stored on LJMU secure data repository
